# Probing Efficient N‐Type Lanthanide Dopants for Mg_3_Sb_2_ Thermoelectrics

**DOI:** 10.1002/advs.202002867

**Published:** 2020-11-13

**Authors:** Jiawei Zhang, Lirong Song, Bo Brummerstedt Iversen

**Affiliations:** ^1^ Center for Materials Crystallography Department of Chemistry and iNANO Aarhus University Aarhus DK‐8000 Denmark

**Keywords:** defect calculations, lanthanide, Mg_3_Sb_2_, n‐type dopants, thermoelectrics

## Abstract

The recent discovery of n‐type Mg_3_Sb_2_ thermoelectrics has ignited intensive research activities on searching for potential n‐type dopants for this material. Using first‐principles defect calculations, here, a systematic computational screening of potential efficient n‐type lanthanide dopants is conducted for Mg_3_Sb_2_. In addition to La, Ce, Pr, and Tm, it is found that high electron concentration (≳10^20^ cm^−3^ at the growth temperature of 900 K) can be achieved by doping on the Mg sites with Nd, Gd, Ho, and Lu, which are generally more efficient than other lanthanide dopants and the anion‐site dopant Te. Experimentally, Nd and Tm are confirmed as effective n‐type dopants for Mg_3_Sb_2_ since doping with Nd and Tm shows higher electron concentration and thermoelectric figure of merit *zT* than doping with Te. Through codoping with Nd (Tm) and Te, simultaneous power factor improvement and thermal conductivity reduction are achieved. As a result, high *zT* values of ≈1.65 and ≈1.75 at 775 K are obtained in n‐type Mg_3.5_Nd_0.04_Sb_1.97_Te_0.03_ and Mg_3.5_Tm_0.03_Sb_1.97_Te_0.03_, respectively, which are among the highest values for n‐type Mg_3_Sb_2_ without alloying with Mg_3_Bi_2_. This work sheds light on exploring promising n‐type dopants for the design of Mg_3_Sb_2_ thermoelectrics.

## Graphical abstract

A systematic computational screening reveals that Nd, Gd, Ho, and Lu are strong candidates as efficient n‐type dopants for Mg_3_Sb_2_ thermoelectrics. Experimentally, Nd and Tm are confirmed as efficient n‐type dopants and high thermoelectric figure of merit *zT* values above 1.6 at 775 K are obtained in n‐type (Nd, Te)‐codoped and (Tm, Te)‐codoped Mg_3_Sb_2_ without Mg_3_Bi_2_ alloying.

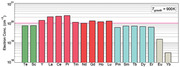

Thermoelectric (TE) materials show great promise in waste heat recovery and solid‐state refrigeration applications since they can directly convert heat into electricity or vice versa purely by solid‐state means.^[^ ^1^, ^2^ ^]^ The performance of TE materials is typically determined by the dimensionless figure of merit *zT* = *α* ^2^ *σT*/*κ*, where *α* is the Seebeck coefficient, *σ* is the electrical conductivity, *T* is the absolute temperature, and *κ* is the total thermal conductivity.

Low‐cost high‐performance materials are required for the widespread application of TE technology. The recently discovered n‐type Mg_3_Sb_2_‐based compound is one such material that shows exceptionally high TE performance with low‐cost and earth‐abundant chemical compositions. The excellent TE performance with *zT* ≈ 1.6 at 725 K was first reported in n‐type Mg_3_Sb_1.5_Bi_0.5_ with Te as an effective electron dopant on the anion sites, where the good TE performance is dominantly attributed to the multiple conducting electron pockets and light conductivity effective mass.^[^ ^3^, ^4^, ^5^ ^]^ Later, many experimental efforts have been made to improve the low‐temperature performance of n‐type Mg_3+_ *_*δ*_*Sb_2−_ *_x_*Bi*_x_*.^[^ ^6^, ^7^, ^8^, ^9^, ^10^, ^11^, ^12^, ^13^ ^]^ Theoretically, accurate electronic structure,^[^ ^14^, ^15^ ^]^ chemical bonding,^[^ ^16^, ^17^, ^18^ ^]^ and phonon transport properties^[^ ^19^, ^20^ ^]^ have been revealed for understanding the outstanding TE properties of n‐type Mg_3_Sb_2_‐based TE materials. Despite many significant studies on n‐type Te‐doped Mg_3_Sb_2_‐based compounds, the optimization of transport properties requires efficient n‐type dopants for the broad tunability of carrier density. For the anion site doping, Se and S also have been reported as effective n‐type dopants for Mg_3_Sb_2_,^[^ ^14^, ^21^ ^]^ whereas they are less efficient than Te due to the higher substitution defect formation energies. Recent defect calculations^[^ ^22^, ^23^, ^24^, ^25^ ^]^ predicted that the group‐3 elements (Sc and Y) as well as several lanthanides including La, Pr, Ce, and Tm are efficient n‐type cation‐site dopants for Mg_3_Sb_2_ and doping with these elements on the Mg sites is able to achieve higher electron concentration than doping with chalcogens on the anion sites. The subsequent experiments^[^ ^24^, ^26^, ^27^, ^28^, ^29^, ^30^, ^31^, ^32^ ^]^ confirmed Sc, Y, La, Pr, and Ce as efficient n‐type dopants on the Mg sites for Mg_3+_ *_*δ*_*Sb_2−_ *_x_*Bi*_x_*. Despite these significant theoretical and experimental efforts on exploring efficient n‐type dopants, the n‐type doping behavior of many other lanthanide dopants in Mg_3_Sb_2_ remains largely unexplored so far.

In this work, we perform first‐principles defect calculations to explore potential n‐type dopants from all lanthanides for Mg_3_Sb_2_ TEs. It is shown that, in addition to La, Ce, Pr, and Tm, several other lanthanides including Nd, Gd, Ho, and Lu are efficient n‐type dopants on the cation sites for Mg_3_Sb_2_. The predicted free electron concentrations for doping with these lanthanide elements in Mg_3_Sb_2_ generally approach or exceed 10^20^ cm^−3^ at the growth temperature of 900 K under the Mg‐rich condition, which are higher than those of doping with the chalcogens. For the experimental validation, we have successfully synthesized n‐type Nd‐doped, Tm‐doped, (Nd, Te)‐codoped, and (Tm, Te)‐codoped Mg_3_Sb_2_ without alloying with Mg_3_Bi_2_. In agreement with the theoretical calculation, the experimental electron concentrations of Nd‐doped and Tm‐doped Mg_3_Sb_2_ samples are indeed higher than those of the Te‐doped ones. Among n‐type Nd‐doped and Tm‐doped samples, optimal *zT* values of 0.16–1.30 and 0.27–1.38 at 300–775 K are obtained in Mg_3.5_Nd_0.04_Sb_2_ and Mg_3.5_Tm_0.03_Sb_2_, respectively. By codoping with the appropriate amount of Nd (Tm) and Te, simultaneous power factor enhancement and thermal conductivity reduction can be achieved. As a result, high *zT* values of ≈1.65 and ≈1.75 are obtained at 775 K, respectively, in Mg_3.5_Nd_0.04_Sb_1.97_Te_0.03_ and Mg_3.5_Tm_0.03_Sb_1.97_Te_0.03_, outperforming any reported n‐type Mg_3_Sb_2_ samples without alloying with Mg_3_Bi_2_.^[^ ^32^, ^33^, ^34^, ^35^ ^]^

The intrinsic point defects under different growth conditions play a crucial role in understanding the doping behavior of Mg_3_Sb_2_. Point defects in Mg_3_Sb_2_ can exist as either electron‐producing donors with positive charges or electron‐capturing acceptors with negative charges, which are indicated by defect plots with positive or negative slopes (see Figure S1 in the Supporting Information), respectively. Because of the high vapor pressure and easy oxidation of Mg, Mg_3_Sb_2_‐based compounds prepared by traditional melt methods are usually Mg‐deficient. Under the Mg‐poor condition, the negatively charged Mg vacancies, acting as the electron‐capturing acceptors, are the dominant native defects showing low defect formation energies within the bulk gap (see Figure S1a in the Supporting Information), which pin the Fermi level close to the valence band maximum (VBM) and thereby make pure Mg_3_Sb_2_ samples persistently p‐type.^[^ ^34^, ^36^ ^]^

In contrast, under the Mg‐rich situation, the defect formation energies of Mg vacancies are notably increased and the Mg interstitials (donor defects) become the dominant native defects (see Figure S1b in the Supporting Information), which means that the Fermi level can move above the middle of the bandgap (mid‐gap) and n‐type conductivity can be achieved. However, the free electron concentration contributed by the positively charged Mg interstitials is very low (in order of ≈10^18^ cm^−3^)^[^ ^22^, ^23^ ^]^ since the compensating acceptor defects Mg vacancies can spontaneously form when the Fermi level is close to the conduction band minimum (CBM), which pin the Fermi level well below the CBM. Thus, the efficient n‐type doping in Mg_3_Sb_2_ requires Mg‐rich growth condition as well as the extrinsic donor defects with sufficiently low formation energies to avoid the electron compensation by the negatively charged Mg vacancies.

To explore potential efficient n‐type dopants for Mg_3_Sb_2_, we investigated the effect of n‐type doping with all lanthanide elements. The formation energies of all the extrinsic defects Ln_Mg_ (i.e., Mg substitution with lanthanides Ln) were calculated using density functional theory (DFT). The results as well as their comparison with the native defects and other extrinsic defects (Te_Sb_, Sc_Mg_, and Y_Mg_) under Mg‐rich conditions are shown in **Figure** **1**a,b and Figures S2–S5 in the Supporting Information. There are two nonequivalent Mg atoms in Mg_3_Sb_2_, where the Mg1 atom occupies the octahedral site between the [Mg_2_Sb_2_]^2−^ slabs while the Mg2 atom resides in the tetrahedral site within the [Mg_2_Sb_2_]^2−^ slabs.^[^ ^37^ ^]^ The formation energies of the extrinsic defects Ln_Mg1_ are lower than those of Ln_Mg2_, which indicates that the lanthanides prefer to substitute Mg1 over Mg2. This might be attributed to the Mg1 atom showing relatively weaker adjacent bonds as well as the larger void space induced by the octahedral site.^[^ ^16^, ^20^ ^]^ All extrinsic defects Ln_Mg1_ generally show no electron compensation by the native defects within the bulk gap since they show much lower formation energies than those of the Mg vacancies and the extrinsic defect Te_Sb_. In defect plots, the slope of the energy line corresponds to the charge state of the defect and the Fermi energy at which the slope changes represents the transition energy level. The substitution defects Ln_Mg1_ generally have +1, 0, and −1 charge states. For n‐type doping, it is favorable to form the donor defect with the charge state +1, while it is detrimental to form the defect with the charge state −1 (or 0) since it can compensate (or limit) the electron concentration.

**Figure 1:**
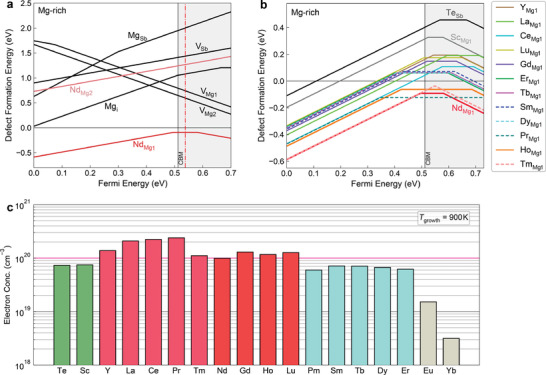
Computational screening of efficient n‐type dopants from lanthanides for Mg_3_Sb_2_. a) Defect formation energies of the Nd doping on the Mg sites as well as the native defects under the Mg‐rich condition. The equilibrium Fermi level at the growth temperature of 900 K is marked with the red dash‐dotted line. b) Defect formation energies of the lanthanide substitution on the Mg1 site located at 1a (0, 0, 0) (denoted as Ln_Mg1_) in comparison with those of the extrinsic defects Te_Mg1_, Sc_Mg1_, and Y_Mg1_in Mg_3_Sb_2_under the Mg‐rich condition. c) Theoretical free electron concentrations at the growth temperature of 900 K for Mg_3_Sb_2_with 15 different n‐type lanthanide dopants. The calculated electron concentrations of the Te, Sc, and Y doping for Mg_3_Sb_2_are used for comparison.

In general, to be efficient n‐type dopants, they not only should have reasonably low formation energies to ensure good solubility but also should show high transition energy levels (+1/0), (0/−1), and (+1/−1) very close to or well above the CBM so that the Fermi level is able to shift close to or above the CBM. It is clear that Eu and Yb are very unfavorable as n‐type dopants since Eu_Mg1_ and Yb_Mg1_ show very low (or deep) (+1/0) transition levels even close to or below the VBM, which pin the Fermi level only slightly above the mid‐gap (see Figure S4e,f in the Supporting Information). In contrast, Y, La, Ce, and Tm are very efficient as n‐type dopants for Mg_3_Sb_2_ because the extrinsic defects Ln_Mg1_ (Ln = Y, La, Ce, and Tm) show high donor transition levels (+1/0) and (+1/−1) well above the CBM (see Figure 1b and Figures S2c–e and S3d in the Supporting Information). Although the extrinsic defect Pr_Mg1_ shows a bit low donor transition level (+1/0) below the CBM, very high transition level (0/−1) located at ≈0.5 eV above the CBM as well as the low formation energy allows the Fermi energy to shift well above the CBM (see Figure 1b and Figure S2f in the Supporting Information), making Pr a very efficient n‐type dopant for Mg_3_Sb_2_. In addition to Y, La, Ce, Pr, and Tm, it is obvious that Nd, Gd, Lu, and Ho are strong candidates as efficient n‐type dopants for Mg_3_Sb_2_ since they show low formation energies as well as the shallow donor transition level (+1/0) close to the CBM (see Figure 1a,b and Figure S3a–c in the Supporting Information). These dopants are typically more efficient than other lanthanides such as Sm, Dy, Er, Pm, and Tb, which, however, show low transition levels (+1/0) and (0/−1) that pin the Fermi level below the CBM (Figure 1b; Figure S4a–d, Supporting Information).

Figure 1c and Figure S6 in the Supporting Information show the calculated free electron concentrations for the efficient n‐type dopants as well as the comparison of free electron concentrations at the growth temperature of 900 K for all lanthanide dopants with those of n‐type Te, Sc, and Y dopants in Mg_3_Sb_2_ under the Mg‐rich condition. Consistent with the results of defect formation energies (see Figure S4 in the Supporting Information), Eu and Yb are largely ineffective as n‐type dopants showing poor free electron concentrations. It is evident that Sm, Dy, Er, Pm, and Tb are a bit less efficient and show lower free electron concentrations than Te for n‐type doping since they show a bit deeper donor levels even though they have lower formation energies. Besides La, Ce, Pr, and Tm that have been confirmed by previous calculations,^[^ ^22^, ^24^, ^25^ ^]^ Nd, Gd, Lu, and Ho, as expected, are more effective as n‐type dopants than Te and doping with these lanthanide elements results in high maximum achievable electron concentrations ≳10^20^ cm^−3^ at 900 K. Among these efficient n‐type dopants, Nd and Tm show the lowest formation energies of the donor defects Nd_Mg1_ (+1) and Tm_Mg1_ (+1) throughout the bulk gap. The predicted maximum achievable free electron concentrations at the growth temperature of 900 K for the potential n‐type dopants Nd, Gd, Lu, and Ho are respectively 9.90 × 10^19^, 1.30 × 10^20^, 1.27 × 10^20^, and 1.17 × 10^20^ cm^−3^, which are slightly lower than those (>2 × 10^20^ cm^−3^) of the n‐type dopants La, Ce, and Pr. Recent experiments^[^ ^24^, ^26^, ^31^ ^]^ have already confirmed La, Ce, and Pr as effective n‐type dopants for Mg_3_Sb_2−_ *_x_*Bi*_x_*, while other predicted efficient n‐type dopants such as Nd, Gd, Lu, Ho, and Tm still require the experimental confirmation.

To verify the theoretical prediction, here, we experimentally examine the potential of Nd and Tm as n‐type dopants for Mg_3_Sb_2_. The Nd‐doped, Tm‐doped, (Nd, Te)‐codoped, and (Tm, Te)‐codoped Mg_3_Sb_2_ bulk samples were prepared by the previously proposed spark plasma sintering (SPS) method^[^ ^30^, ^38^ ^]^ followed by annealing in the Mg‐rich condition.^[^ ^11^ ^]^ According to our previous work,^[^ ^30^ ^]^ the specific amount of excess Mg (Mg_3.5_) is used to compensate for the evaporation loss of Mg so that the as‐SPS‐pressed pellets show no Mg deficiency. All as‐synthesized bulk samples are dense with relative densities larger than 95% (Table S1, Supporting Information). Powder X‐ray diffraction (PXRD) patterns confirm that all samples are virtually phase pure with a very small amount of MgO (see **Figure** **2**a,b and Figures S7 and S8 in the Supporting Information). The Nd‐doped sample Mg_3.5_Nd_0.04_Sb_2_ and all (Nd, Te)‐codoped samples show a small amount of secondary phase, which can be indexed to the NdSb cubic phase (space group: $\mathrm{Fm}\bar{3}m$). Similarly, a small amount of secondary phase TmSb is found in several Mg_3.5_Tm*_y_*Sb_2−_ *_x_*Te*_x_* samples (*y* = 0.03–0.04, *x* = 0 and 0.03). For Nd‐doped samples Mg_3.5_Nd*_y_*Sb_2_, the lattice parameters show a gradually increasing trend as the fraction *y* increases from 0.01 to 0.03 (see Table S2 in the Supporting Information). This can be understood by the larger ionic radius of Nd^3+^ than that of Mg^2+^. On the other hand, the lattice parameters decrease when the fraction *y* increases from 0.03 to 0.04, which is likely induced by the formation of the secondary phase NdSb. A similar trend in the lattice parameters is found in Tm‐doped samples (Table S2, Supporting Information), which can be understood in the same manner.

**Figure 2:**
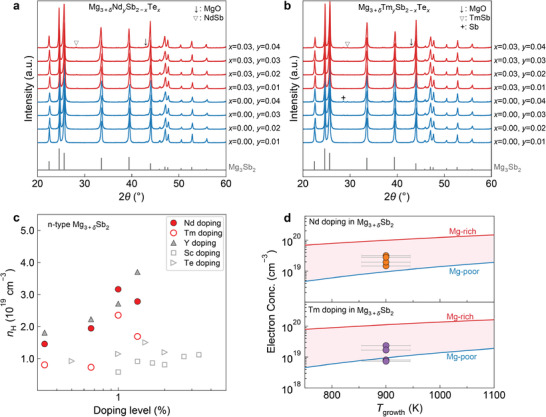
PXRD patterns of the bulk samples with the nominal compositions a) Mg_3+_ *_*δ*_*Nd*_y_*Sb_2−_ *_x_*Te*_x_* and b) Mg_3+_ *_*δ*_*Tm*_y_*Sb_2−_ *_x_*Te*_x_*(*δ*= 0.5, *x*= 0 and 0.03, *y*= 0.01–0.04) measured before property measurements. c) Experimental Hall carrier concentration as a function of the doping level for the n‐type doping with Nd and Tm comparing with doping with other elements (Y, Sc,^[^ ^30^ ^]^ and Te^[^ ^30^ ^]^) in Mg_3+_ *_*δ*_*Sb_2_without the Mg_3_Bi_2_alloying. The data of Nd‐, Tm‐, and Y‐doped samples are from this work. d) Predicted free electron concentration as a function of the growth temperature of the n‐type doping with Nd and Tm in Mg_3_Sb_2_under the Mg‐rich and Mg‐poor conditions. The orange and purple solid points represent the experimental data of n‐type Nd‐doped and Tm‐doped Mg_3+_ *_*δ*_*Sb_2_, respectively. The growth temperature of the experimental data is set at 900 K with an uncertainty of 5%.

The energy dispersive X‐ray spectroscopy (EDS) elemental mapping of the Mg_3.5_Nd_0.03_Sb_1.97_Te_0.03_ sample shows that the sample is virtually homogeneous with nearly uniform distributions of the constituent elements (Figure S9, Supporting Information). The chemical composition measured by scanning electron microscopy (SEM)‐EDS reveals that the actual Mg content of the Mg_3.5_Nd_0.03_Sb_1.97_Te_0.03_ sample increases from Mg_3.05_ to Mg_3.3_ after annealing in the Mg‐rich condition in an Argon atmosphere (Table S3, Supporting Information). The evolution of the PXRD patterns confirms that the amount of MgO shows nearly no change after annealing (Figure S8, Supporting Information), which indicates that the 10% excess Mg after annealing should not exist dominantly as the MgO secondary phase at the grain boundaries. The increase in cell parameters after annealing in the Mg‐rich condition could be an indication of the excess Mg partially entering the interstitial sites of the structure (Table S4, Supporting Information). However, only a small proportion of the excess Mg might exist as the Mg interstitials within the structure due to the small solubility.^[^ ^34^ ^]^ Therefore, the excess Mg should be dominantly contributed by the elemental Mg that diffuses into the grain boundaries of the sample during the Mg‐rich annealing, which is confirmed by the elemental Mg peaks in the PXRD pattern of the Mg_3.5_Nd_0.03_Sb_1.97_Te_0.03_ sample right after the annealing (Figure S8, Supporting Information).

For the better comparison of the doping effect on the carrier concentration and mobility in n‐type Mg_3_Sb_2_, several Y‐doped samples Mg_3.5_Y*_y_*Sb_2_ (*y* = 0.01–0.04) were also prepared using the same method as for Nd‐doped samples and the corresponding experimental results are shown in Figures S10–S13 in the Supporting Information. With the experimental data of the Nd‐, Tm‐, and Y‐doped samples, we can compare the results with those of the reported Te‐ and Sc‐doped samples prepared using a similar synthesis method.^[^ ^30^ ^]^ It is found that n‐type Nd‐doped and Tm‐doped Mg_3_Sb_2_ samples show slightly lower electron concentrations than those of the Y‐doped samples but higher electron concentrations than those of Sc‐doped and Te‐doped samples (see Figure 2c), which is consistent with the theoretical calculation. The room temperature electron concentrations of n‐type Mg_3.5_Nd*_y_*Sb_2_ samples vary from 1.46 × 10^19^ (*y* = 0.01) to 3.17 × 10^19^ cm^−3^ (*y* = 0.03), while the electron concentrations vary from 7.32 × 10^18^ to 2.36 × 10^19^ cm^−3^ in Mg_3.5_Tm*_y_*Sb_2_ samples. Nearly all experimental electron concentration data points of Nd‐ and Tm‐doped samples fall exactly within the pink region shown in Figure 2d with the predicted free electron concentration at the Mg‐rich and Mg‐poor condition as the upper and lower bound, respectively. Further improvement of the electron concentration of Nd‐doped and Tm‐doped samples can be expected through reducing the bandgap via alloying with Mg_3_Bi_2_.

**Figure** **3** shows the temperature‐dependent electron mobility of all samples. Although the Nd‐doped samples have been sintered at a high temperature of 800 °C followed by annealing at 615 °C under the Mg‐rich environment^[^ ^11^ ^]^ (see the Experimental Section) to minimize the grain boundary scattering, the electron mobility of Nd‐doped Mg_3_Sb_2_ samples still shows a temperature dependence of *T* ^1.5^ at low temperatures. This is very different from the Y‐doped, Sc‐doped,^[^ ^30^ ^]^ or Te‐doped^[^ ^30^ ^]^ samples synthesized using a similar method, which show dominant acoustic phonon scattering at low temperatures (Figure S14, Supporting Information). The increasing temperature dependence of the mobility at low temperatures results in poor room‐temperature electron mobility (≈20.8–32.3 cm^2^ V^−1^ s^−1^) of Nd‐doped samples in comparison with those of Te‐doped samples (≈51.0–73.8 cm^2^ V^−1^ s^−1^).^[^ ^30^ ^]^ By codoping with Nd and Te, acoustic phonon scattering becomes dominant throughout the entire temperature range as indicated by the temperature dependence of the mobility following T^−p^ (1  ≤  p  ≤  1.5). As a result, the (Nd, Te)‐codoped Mg_3_Sb_2_ samples show enhanced room‐temperature electron mobility values of ≈38.4–54.6 cm^2^ V^−1^ s^−1^, which, however, are still lower than those of many reported Te‐doped Mg_3_Sb_2_ samples.^[^ ^30^ ^]^

**Figure 3:**
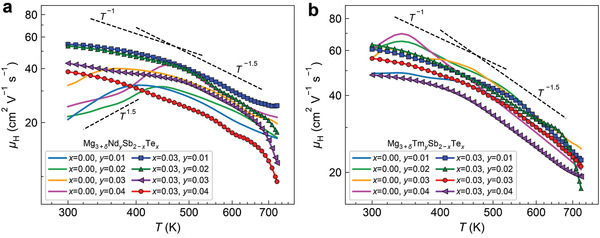
Temperature dependence of the Hall mobility of n‐type a) Mg_3+_ *_*δ*_*Nd*_y_*Sb_2−_ *_x_*Te*_x_*and b) Mg_3+_ *_*δ*_*Tm*_y_*Sb_2−_ *_x_*Te*_x_*(*δ*= 0.5).

The low carrier mobilities in Nd‐doped samples in comparison with Y‐, Sc‐, and Te‐doped samples (see Figure 3a and Figures S14 and S15a in the Supporting Information) may be induced by the impurity states near the CBM introduced by the Nd doping. The low carrier mobilities at low temperatures in Nd‐doped samples may be attributed to the impurity band conduction (Note S1, Supporting Information).^[^ ^39^ ^]^ By comparing the calculated density of states (DOS) of the defect structure Mg_53_Nd_1_Sb_36_ (Mg_2.944_Nd_0.056_Sb_2_) with the perfect structure Mg_54_Sb_36_ (Mg_3_Sb_2_), it is found that the f states of the impurity atom Nd exist largely below the CBM within the bulk gap (Figure S15b, Supporting Information). Moreover, the Nd f states show hybridization with the electronic states (Mg 3s states) of the near‐edge conduction bands resulting in the enhanced DOS of the CBM. The relatively localized feature of the f states below the CBM as well as the enhanced DOS (effective mass) of the CBM induced by the hybridization of the f states with the near‐edge electronic states is likely the origin of low electron mobility in Nd‐doped Mg_3_Sb_2_. The appearance of the f states below the CBM as well as their hybridization with the conduction band in Nd‐doped samples can be attributed to the relatively small energy separation between the atomic orbital levels of the Nd f states and Mg 3s states. Similarly, the above mechanism may be used to explain the low electron mobilities in previously reported La‐, Pr‐, and Ce‐doped Mg_3_Sb_2−_ *_x_*Bi*_x_* samples^[^ ^24^, ^26^, ^31^ ^]^ because of the small energy separation between the atomic orbital energies of the f states of the dopants (La, Pr, and Ce) and 3s states of Mg (see Table S5 in the Supporting Information).

In comparison with Nd‐doped samples, Tm‐doped Mg_3_Sb_2_ samples generally show clearly higher electron mobilities especially at low temperatures. Room‐temperature electron mobilities (≈48.8–60.7 cm^2^ V^−1^ s^−1^) of Tm‐doped samples are comparable to those of many reported Te‐doped Mg_3_Sb_2_ samples.^[^ ^30^ ^]^ This is because that the f states of the Tm dopant mainly contributed to the electronic states of the valence bands (rather than within the bulk gap and conduction bands) in Tm‐doped Mg_3_Sb_2_ due to the much lower atomic orbital energies of the Tm f states (see Figure S16 and Table S5 in the Supporting Information).

**Figures** **4** and **5** show the TE properties of n‐type Nd‐doped, Tm‐doped, (Nd, Te)‐codoped, and (Tm, Te)‐codoped Mg_3_Sb_2_ samples. The Seebeck coefficient and electrical resistivity of all samples show typical degenerate semiconductor behaviors with increasing temperature dependence at elevated temperatures. The resistivity of several Nd‐doped and Tm‐doped samples shows a clear decreasing trend with increasing temperature at low temperatures, which is induced by the increasing temperature dependence of the mobility. Unlike the reported Te‐doped samples showing clear hysteresis,^[^ ^4^ ^]^ the resistivity data of Mg_3.5_Nd*_y_*Sb_2_ and Mg_3.5_Tm*_y_*Sb_2_ samples show virtually no hysteresis upon thermal cycling (Figures S17 and S18, Supporting Information), indicating the enhanced stability and good repeatability. With increasing *y* from 0.01 to 0.03 in Mg_3.5_Nd*_y_*Sb_2_ and Mg_3.5_Tm*_y_*Sb_2_, the resistivity and absolute values of the Seebeck coefficient decreases as the electron concentration increases (see Figures 2c, 4a,b, and 5a,b). With the Nd and Te codoping, the resistivity can be effectively reduced in Mg_3.5_Nd*_y_*Sb_1.97_Te_0.03_ mainly due to the enhanced electron mobility. As a result of the reduced resistivity and moderate Seebeck coefficient, the power factor of n‐type (Nd, Te)‐codoped samples exhibits a significant enhancement over the Nd‐doped samples (Figure 4c). Because of lower resistivities induced by higher electron mobilities, the power factors of Tm‐doped samples are generally higher than those of Nd‐doped samples. With the proper Tm and Te codoping, the power factors can be further improved up to ≈12.5–16.0 µW cm^−1^ K^−2^ within 300–725 K in Mg_3.5_Tm_0.02_Sb_1.97_Te_0.03_ (Figure 5c). The total thermal conductivity values of Nd‐doped and Tm‐doped samples are generally lower than those of the reported Te‐doped^[^ ^30^ ^]^ samples prepared using the same method (Figures 4d and 5d). By codoping with the appropriate amount of Nd (Tm) and Te, the thermal conductivity is effectively reduced to 0.54 W m^−1^ K^−1^ (0.53 W m^−1^ K^−1^) at 775 K in Mg_3.5_Nd_0.04_Sb_1.97_Te_0.03_ (Mg_3.5_Tm_0.03_Sb_1.97_Te_0.03_).

**Figure 4:**
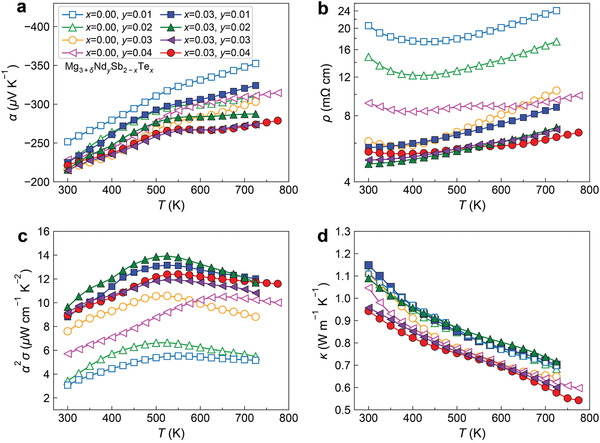
a) Temperature‐dependent Seebeck coefficient, b) electrical resistivity, c) power factor, and d) total thermal conductivity of n‐type Mg_3+_ *_*δ*_*Nd*_y_*Sb_2−_ *_x_*Te*_x_*(*δ*= 0.5).

**Figure 5:**
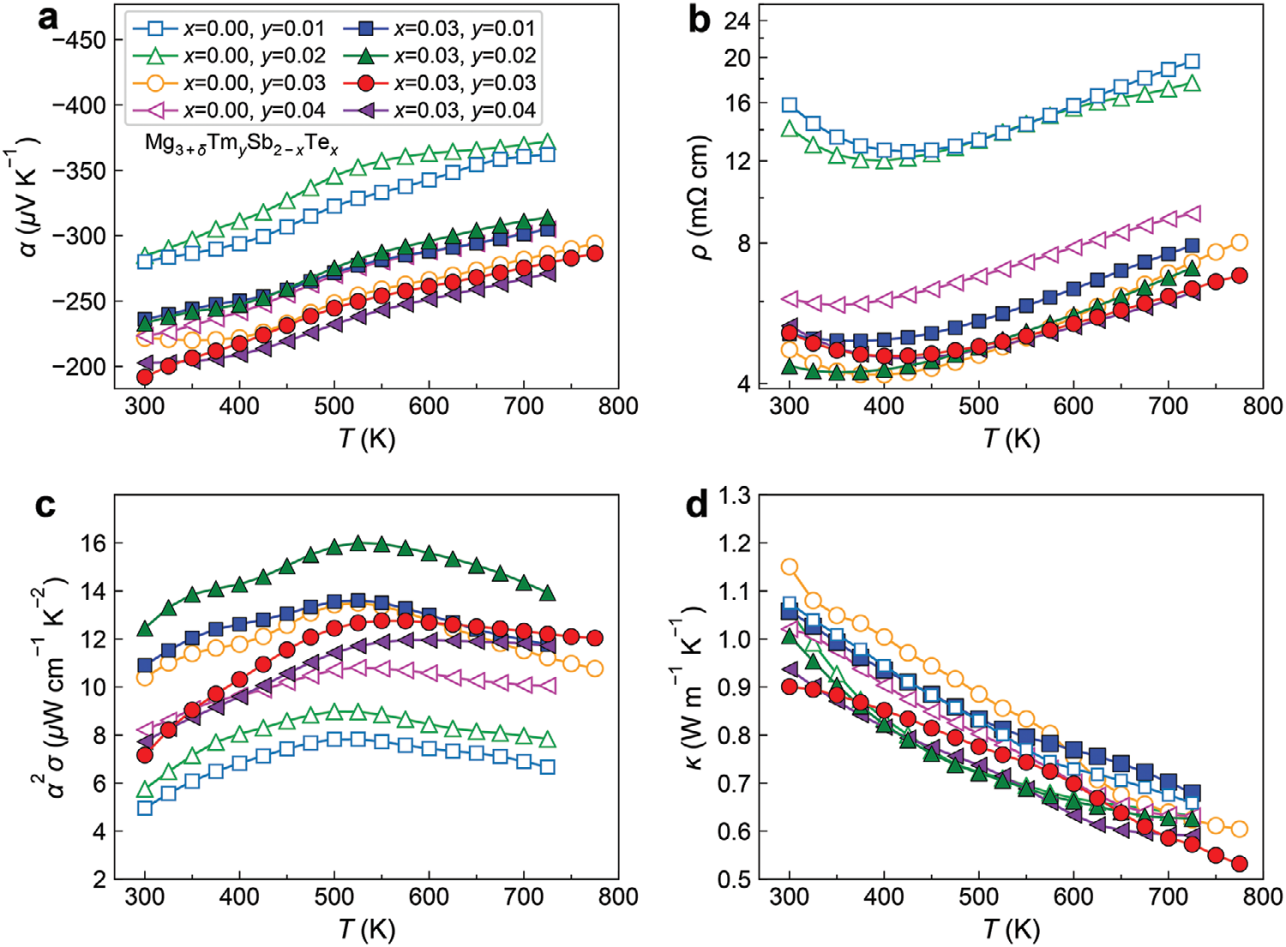
a) Temperature‐dependent Seebeck coefficient, b) electrical resistivity, c) power factor, and d) total thermal conductivity of n‐type Mg_3+_ *_*δ*_*Tm*_y_*Sb_2−_ *_x_*Te*_x_*(*δ*= 0.5).

As shown in **Figure** **6**a, for Mg_3.5_Nd*_y_*Sb_2_ samples, an optimal *zT* of 1.30 at 775 K is obtained in the sample with *y* = 0.04, which is superior to the reported Te‐doped Mg_3_Sb_2_ samples.^[^ ^30^, ^34^ ^]^ In comparison with Nd‐doped samples, a higher *zT* of 0.27–1.38 at 300–775 K is achieved in n‐type Mg_3.5_Tm_0.03_Sb_2_ (see Figure 6b), which outperforms other n‐type singly doped Mg_3_Sb_2_ samples.^[^ ^30^, ^32^, ^33^, ^34^, ^35^ ^]^ This confirms Nd and Tm as effective n‐type dopants for Mg_3_Sb_2_, which is in agreement with the theoretical prediction. Owing to the combined effect of the enhanced power factor and low thermal conductivity, the (Nd, Te)‐codoped and (Tm, Te)‐codoped samples generally show strongly enhanced *zT* values in comparison with those of singly doped samples. Peak *zT* values of 1.65 and 1.75 at 775 K are achieved, respectively, in Mg_3.5_Nd_0.04_Sb_1.97_Te_0.03_ and Mg_3.5_Tm_0.03_Sb_1.97_Te_0.03_, which are higher than those of previously reported n‐type Mg_3_Sb_2_ samples^[^ ^30^, ^32^, ^33^, ^34^, ^35^ ^]^ without alloying with Mg_3_Bi_2_ (see Figure 6c). Considering most of the previous reports^[^ ^32^, ^33^, ^34^, ^35^ ^]^ showing *zT* smaller than unity in n‐type Mg_3_Sb_2_ without the Mg_3_Bi_2_ alloying, the high *zT* reported in this work represents a great advance for the development of high‐performance n‐type Mg_3_Sb_2_ without alloying with Mg_3_Bi_2_.

**Figure 6:**
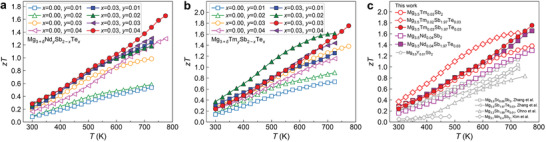
Temperature dependence of the figure of merit *zT* of n‐type a) Mg_3+_ *_*δ*_*Nd*_y_*Sb_2−_ *_x_*Te*_x_* and b) Mg_3+_ *_*δ*_*Tm*_y_*Sb_2−_ *_x_*Te*_x_*(*δ*= 0.5). c) *zT* values of n‐type Mg_3.5_Nd_0.04_Sb_2_, Mg_3.5_Nd_0.04_Sb_1.97_Te_0.03_, Mg_3.5_Tm_0.03_Sb_2_, Mg_3.5_Tm_0.02_Sb_1.97_Te_0.03_, and Mg_3.5_Tm_0.03_Sb_1.97_Te_0.03_ in comparison with those of the n‐type Y‐doped (this work), reported Te‐doped,^[^ ^30^, ^34^ ^]^ Sc‐doped,^[^ ^30^ ^]^ and Mn‐doped^[^ ^33^ ^]^ Mg_3+_ *_*δ*_*Sb_2_ without alloying with Mg_3_Bi_2_.

In summary, we have conducted a complete computational screening of promising n‐type dopants from all lanthanide elements for Mg_3_Sb_2_ thermoelectrics. In addition to La, Ce, Pr, and Tm that have been previously predicted, it is found that Nd, Gd, Lu, and Ho are strong candidates as efficient n‐type cation‐site dopants for Mg_3_Sb_2_ and the maximum achievable free electron concentrations for doping with these lanthanides reach ≳10^20^ cm^−3^ at the growth temperature of 900 K, which is higher than that of the anion‐site doping with Te. For the experimental validation, we report successful n‐type doping in Mg_3_Sb_2_ with Nd and Tm. In agreement with our theoretical prediction, Nd and Tm are experimentally confirmed as efficient n‐type dopants for Mg_3_Sb_2_, which indeed show higher experimental electron concentration than n‐type doping with Te. For n‐type Nd‐doped and Tm‐doped Mg_3_Sb_2_ samples, we obtain high *zT* values of 1.30 and 1.38 at 775 K, respectively. With the (Nd, Te)‐codoping and (Tm, Te)‐codoping in Mg_3_Sb_2_, we achieve optimal *zT* values of 1.65 and 1.75 at 775 K, respectively, superior to any reported n‐type Mg_3_Sb_2_ samples without alloying with Mg_3_Bi_2_. We can expect further improvements in TE performance through optimizing carrier concentration as well as reducing thermal conductivity via alloying with Mg_3_Bi_2_. This work provides insight into the exploration of potential effective n‐type dopants for the development of Mg_3_Sb_2_ TE materials using an integrated computational and experimental approach.

## Experimental Section

##### Theoretical Calculations

All calculations in this work were conducted using the projector augmented wave method^[^ ^40^, ^41^ ^]^ in Vienna Ab initio Simulation Package (VASP)^[^ ^42^ ^]^. The defect calculations were conducted in a 3 × 3 × 2 supercell with 90 atoms using the hybrid functional HSE06.^[^ ^43^ ^]^ A mixing parameter of 25% was used for the calculations with the HSE06 functional. The energy and Hellmann–Feynman force convergence criteria were 10^−4^ eV and 0.01 eV Å^−1^, respectively. An energy cutoff of 400 eV was used for the plane‐wave expansions. A Γ‐centered 2 × 2 × 2 *k* mesh was applied for the crystal structure optimization and the total energy calculation. For the optimization of defect structures, the lattice parameters were fixed at the optimized values of the perfect supercell and all atomic positions in the defect supercell were allowed to be fully relaxed into their equilibrium positions. The symmetry was switched off during the structural relaxation. Spin polarization was included in all defect calculations. Due to the slow convergence of structural relaxations, the extrinsic defects Sm_Mg_, Dy_Mg_, Tb_Mg_, and Er_Mg_ were calculated using the Perdew‐Burke‐Ernzerhof (PBE) functional^[^ ^44^ ^]^ and a Γ‐centered 4 × 4 × 4 *k* mesh with the band edge shifting correction (Δ*E*_VBM_ = −0.24 eV, Δ*E*_CBM_ = 0.18 eV) so that the bandgap is corrected to be equal to the result (0.51 eV) with the HSE06 functional. The density of states of the relaxed defect supercell Mg_53_Nd_1_Sb_36_ (Mg_53_Tm_1_Sb_36_) with one Nd (Tm) atom substituting on the Mg1 site and the bulk supercell Mg_54_Sb_36_ shown in Figures S15b and S16 in the Supporting Information were calculated with the TB‐mBJ potential^[^ ^45^ ^]^ and a Γ‐centered 6 × 6 × 6 *k* mesh.

For a point defect d with a charge state *q*_d_, the formation energy can be calculated using^[^ ^46^, ^47^ ^]^

(1) $$\begin{array}{c} \Delta E_{f}^{d}=E_{\mathrm{tot}}^{d}-E_{\mathrm{tot}}^{\mathrm{bulk}}-\sum_{i}n_{i}\mu_{i}+q_{d}\left(\varepsilon_{F}+E_{V}\right)+E_{\mathrm{corr}} \end{array}$$

Here, $E_{\mathrm{tot}}^{d}$ is the total energy of the supercell with the point defect d, and $E_{\mathrm{tot}}^{\mathrm{bulk}}$ is the total energy of the perfect supercell. *n_i_* represents the number of atoms of type *i* that is added to (*n_i_* > 0) or removed from (*n_i_* < 0) the supercell when the point defect is formed, and *µ_i_* represents the atomic chemical potentials of these species. *ε*_F_ is the Fermi level, which is referenced to the valence‐band maximum *E*_V_ of the perfect bulk structure. *E*_corr_is the correction term induced by the finite size of charged supercells. Here, the image charge and potential alignment corrections for charged defects were taken into account.^[^ ^48^ ^]^

In Mg_3_Sb_2_, the chemical potentials of Mg and Sb must fulfill the thermodynamic stability condition: 3Δ*μ*_Mg_ + 2Δ*μ*_Sb_ = 5Δ*H*_f_(Mg_3_Sb_2_). To avoid the precipitations of the constituent elements, the chemical potentials should satisfy the condition: $\Delta \mu_{\alpha }=\mu_{\alpha }-\mu_{\alpha }^{0}\leq 0$, in which $\mu_{\alpha }^{0}$ denotes the total energy per atom in the pure bulk crystal of the element *α*. Under the Mg‐rich condition, the chemical potentials of Q (Q = Y, Sc, La, Ce, Pr, Pm, Nd, Sm, Gd, Tb, Dy, Ho, Er, Tm, and Lu) are limited by the formation energies of the competing phases QSb (Δ*μ*_Q_ + Δ*μ*_Sb_ ≤ 2Δ*H*_f_(QSb)), the chemical potentials of M (M = Eu and Yb) are constrained by M_5_Sb_3_ (5Δ*μ*_M_ + 3Δ*μ*_Sb_ ≤ 8Δ*H*_f_(M_5_Sb_3_)), and the chemical potential of Te is limited by MgTe (Δ*μ*_Mg_ + Δ*μ*_Te_ ≤ 2Δ*H*_f_(MgTe)). For the calculations of defect formation energies of Q_Sb_ (Q = Nd, Gd, Ho, Lu, and Tm) under the Sb‐rich condition (Δ*μ*_Sb_ =  0), the chemical potentials of Q are also limited by the formation energies of the competing phases QSb. For PmSb (hypothetical), the structure is assumed to be the same as NdSb. Formation energies of the bulk phase and competing phases are in units of eV per atom.

Under the thermodynamic equilibrium, the Fermi energy at different growth temperatures can be obtained by self‐consistently solving the charge neutrality equation^[^ ^36^, ^46^ ^]^

(2) $$\begin{array}{c} n_{h}-n_{e}+\sum_{d}q_{d}c_{d}=0 \end{array}$$

Taking the approximation of the free energy by the defect formation energy, the defect concentration can be calculated using^[^ ^46^, ^47^ ^]^

(3) $$\begin{array}{c} c_{d}=c_{0}^{d}e^{-\Delta E_{f}^{d}/k_{B}T} \end{array}$$

where $c_{0}^{d}$ is the concentration of defect sites for the point defect d, and *k*_B_ is the Boltzmann constant. In the dilute limit, the electronic structure is assumed to be the same as that of the perfect neutral bulk cell. The numbers of holes and electrons are calculated, respectively, by

(4) $$\begin{array}{c} n_{h}=\int_{-\infty }^{E_{V}}N(\varepsilon )\left[1-f\left(\varepsilon ,\varepsilon_{F}\right)\right]d\varepsilon \end{array}$$

(5) $$\begin{array}{c} n_{e}=\int_{E_{C}}^{\infty }N(\varepsilon )f\left(\varepsilon ,\varepsilon_{F}\right)d\varepsilon \end{array}$$

*E*_C_ is the energy of the conduction band minimum. *f*(*ε*, *ε*_F_) is the Fermi–Dirac distribution. For *N*(*ε*), the accurate density of states of the Mg_3_Sb_2_ unit cell calculated by the HSE06 functional with the tetrahedron method and a dense Γ‐centered 16 × 16 × 10 *k* mesh was used. For solving the equilibrium Fermi level of the lanthanide (Ln) doping in Mg_3_Sb_2_ with Equation (2), the extrinsic defects Ln_Mg1_ and Ln_Mg2_ together with all intrinsic defects with different charge states was considered. The relatively negative formation energies of Eu_Mg1_, Yb_Mg1_, and Pm_Mg1_ might be due to some unknown competing phases that have not been taken into account during the calculations. The limitation of the DFT calculations for the lanthanides with f‐electrons, described in Note S2 in the Supporting Information, might also cause some uncertainties. Therefore, we should focus on the relative trend and transition levels for defect calculations with lanthanide dopants.

##### Sample Synthesis

Stoichiometric amounts of high‐purity Mg powder (99.8%, −325 mesh, Alfa Aesar), Sb powder (99.5%, −325 mesh, Chempur), Nd powder (99.9%, −200 mesh, Chempur), Tm powder (99.9%, −200 mesh, Chempur), Te powder (99.99%, −325 mesh, Chempur), and Y powder (99.9%, −200 mesh, Chempur) were weighed according to the nominal compositions of Mg_3+_ *_*δ*_*Nd*_y_*Sb_2−_ *_x_*Te*_x_* (*δ* = 0.5, *y* = 0.01–0.04, *x* = 0 and 0.03), Mg_3+_ *_*δ*_*Tm*_y_*Sb_2−_ *_x_*Te*_x_* (*δ* = 0.5, *y* = 0.01–0.04, *x* = 0 and 0.03), and Mg_3+_ *_*δ*_*Y*_y_*Sb_2_ (*δ* = 0.5, *y* = 0.01–0.04), and mixed in a mixer (SpectroMill, Chemplex Industries, Inc.) without using ball pestles for 15 min. The excess Mg here was typically used for the purpose to compensate for the evaporation loss of Mg during the high‐temperature synthesis. The mixed powders were loaded into a 12.7 mm diameter graphite die protected with the graphite paper. The above processes were conducted inside an argon‐filled glove box. Afterward, the mixed powders loaded in the graphite die were immediately sintered and consolidated in dynamic vacuum under a uniaxial pressure of 60 MPa using an SPS‐515S instrument (SPS Syntex Inc., Japan). The SPS process was conducted at 673 K with a 10 min dwell and then heating to 1073 K with another 10 min dwell. Following the SPS, the annealing of the pellets was conducted in the Mg‐rich environment similar to the procedure proposed by Wood et al.^[^ ^11^ ^]^ The as‐pressed pellets together with Mg turnings (99.9%, Chempur) were placed in a glassy carbon crucible with a lid and annealed in Ar flow at 888 K for 3 days. After annealing in the Mg‐rich condition, the pellets were taken out and slightly polished before property measurements.

##### Structure Characterization

The phase purity of the bulk samples was checked by the PXRD measured on a Rigaku Smartlab in the Bragg–Brentano (BB) geometry using a Cu K*α*_1_ source. The lattice parameters were determined by the Le Bail method^[^ ^49^ ^]^ using the JANA2006^[^ ^50^ ^]^ program. The microstructure, elemental distribution, and chemical composition were characterized using a scanning electron microscope (FEI Nova NanoSEM 600) equipped with an energy dispersive spectrometer.

##### TE Transport Property Measurements

Measurements of electrical resistivity *ρ* and Hall coefficient (*R*_H_) were implemented using the van der Pauw method^[^ ^51^ ^]^ in dynamic vacuum (<10^−4^ mbar) under a magnetic field up to 1.25 T.^[^ ^52^, ^53^ ^]^ Hall carrier concentration (*n*_H_) and mobility (*μ*_H_) were evaluated respectively by 1/*eR*_H_ and *R*_H_/*ρ*, where *e* is the elementary charge. The Hall measurements of the pellets were carried out with the heating and cooling processes for 3 thermal cycles (300–725 K), in which the final stabilized cycle is used for discussion in the main text. The temperature‐dependent Seebeck coefficients of the pellets were then measured with the slope method using chromel‐niobium thermocouples in dynamic vacuum (<10^−4^ mbar) on an in‐house system,^[^ ^53^ ^]^ whose geometry is similar to the one reported by Iwanaga et al.^[^ ^54^ ^]^ The thermal diffusivity (*D*) was measured using the laser flash method (Netzsch, LFA457). The density (*d*) was estimated from the mass and volume of the pellets. Thermal conductivity was then determined using the formula *κ*  =  *d* *DC*_P_. In the main text, the thermal conductivity and *zT* were evaluated using the heat capacity (*C*_P_) (see Figure S19 in the Supporting Information) calculated with the polynomial equation proposed by Agne et al.^[^ ^55^ ^]^ For comparison, the thermal conductivity and *zT* estimated with the heat capacity (*C*_P_) from the LFA457 setup using a Pyroceram 9606 standard sample are shown in Figures S20 and S21 in the Supporting Information. For simplicity, here, the cooling curves of transport properties were adopted for discussion in the main text. The Seebeck coefficient, electrical resistivity, and thermal conductivity typically have the measurement uncertainties of 7%, 5%, and 7%, respectively.^[^ ^56^, ^57^ ^]^ The uncertainty for *zT* is about 20%.

## Conflict of Interest

The authors declare no conflict of interest.

## Supporting information

Supporting Information

## References

[1] G. S. Nolas , J. Sharp , H. J. Goldsmid , Thermoelectrics: Basic Principles and New Materials Developments, Springer, New York 2001.

[2] a) G. J. Snyder , E. S. Toberer , Nat. Mater. 2008, 7, 105; 18219332 10.1038/nmat2090

[3] S. H. Pedersen, Master's Thesis, Thermoelectric properties of Zintl compounds Mg3Sb2−xBix, Aarhus University, 2012 http://chem.au.dk/fileadmin/cmc.chem.au.dk/pictures new homepage/Pedersen S H ‐ Thermoelectric Properties of Zintl Compounds Mg3Sb2-xBix.pdf.

[4] J. Zhang , L. Song , S. H. Pedersen , H. Yin , L. T. Hung , B. B. Iversen , Nat. Commun. 2017, 8, 13901. 28059069 10.1038/ncomms13901 PMC5227096

[5] H. Tamaki , H. K. Sato , T. Kanno , Adv. Mater. 2016, 28, 10182. 27690372 10.1002/adma.201603955

[6] J. Shuai , J. Mao , S. Song , Q. Zhu , J. Sun , Y. Wang , R. He , J. Zhou , G. Chen , D. J. Singh , Z. Ren , Energy Environ. Sci. 2017, 10, 799.

[7] J. Mao , J. Shuai , S. W. Song , Y. X. Wu , R. Dally , J. W. Zhou , Z. H. Liu , J. F. Sun , Q. Y. Zhang , C. dela Cruz , S. Wilson , Y. Z. Pei , D. J. Singh , G. Chen , C. W. Chu , Z. F. Ren , Proc. Natl. Acad. Sci. USA 2017, 114, 10548. 28923974 10.1073/pnas.1711725114 PMC5635921

[8] X. Chen , H. J. Wu , J. Cui , Y. Xiao , Y. Zhang , J. Q. He , Y. Chen , J. Cao , W. Cai , S. J. Pennycook , Z. H. Liu , L. D. Zhao , J. H. Sui , Nano Energy 2018, 52, 246.

[9] T. Kanno , H. Tamaki , H. K. Sato , S. D. Kang , S. Ohno , K. Imasato , J. J. Kuo , G. J. Snyder , Y. Miyazaki , Appl. Phys. Lett. 2018, 112, 033903.

[10] J. J. Kuo , S. D. Kang , K. Imasato , H. Tamaki , S. Ohno , T. Kanno , G. J. Snyder , Energy Environ. Sci. 2018, 11, 429.

[11] M. Wood , J. J. Kuo , K. Imasato , G. J. Snyder , Adv. Mater. 2019, 31, 1902337. 10.1002/adma.201902337 31273874

[12] K. Imasato , S. D. Kang , G. J. Snyder , Energy Environ. Sci. 2019, 12, 965.

[13] J. Mao , H. Zhu , Z. Ding , Z. Liu , G. A. Gamage , G. Chen , Z. Ren , Science 2019, 365, 495. 31320557 10.1126/science.aax7792

[14] J. Zhang , L. Song , A. Mamakhel , M. R. V. Jørgensen , B. B. Iversen , Chem. Mater. 2017, 29, 5371.

[15] J. Zhang , B. B. Iversen , J. Appl. Phys. 2019, 126, 085104.

[16] J. Zhang , L. Song , M. Sist , K. Tolborg , B. B. Iversen , Nat. Commun. 2018, 9, 4716. 30413702 10.1038/s41467-018-06980-x PMC6226478

[17] C. Zheng , R. Hoffmann , R. Nesper , H. G. Von Schnering , J. Am. Chem. Soc. 1986, 108, 1876.

[18] X. Sun , X. Li , J. Yang , J. Xi , R. Nelson , C. Ertural , R. Dronskowski , W. Liu , G. J. Snyder , D. J. Singh , W. Zhang , J. Comput. Chem. 2019, 40, 1693. 30889285 10.1002/jcc.25822

[19] W. Peng , G. Petretto , G. M. Rignanese , G. Hautier , A. Zevalkink , Joule 2018, 2, 1879.

[20] J. Zhang , L. Song , B. B. Iversen , npj Comput. Mater. 2019, 5, 76.

[21] J. Zhang , L. Song , K. A. Borup , M. R. V. Jørgensen , B. B. Iversen , Adv. Energy Mater. 2018, 8, 1702776.

[22] P. Gorai , B. R. Ortiz , E. S. Toberer , V. Stevanovic , J. Mater. Chem. A 2018, 6, 13806. 10.1039/C8TA07539E PMC7121276 32257213

[23] P. Gorai , E. S. Toberer , V. Stevanović , J. Appl. Phys. 2019, 125, 025105.

[24] J. Li , F. Jia , S. Zhang , S. Zheng , B. Wang , L. Chen , G. Lu , L. Wu , J. Mater. Chem. A 2019, 7, 19316.

[25] J. Li , S. Zhang , S. Zheng , Z. Zhang , B. Wang , L. Chen , G. Lu , J. Phys. Chem. C 2019, 123, 20781.

[26] K. Imasato , M. Wood , J. J. Kuo , G. J. Snyder , J. Mater. Chem. A 2018, 6, 19941.

[27] S. W. Song , J. Mao , M. Bordelon , R. He , Y. M. Wang , J. Shuai , J. Y. Sun , X. B. Lei , Z. S. Ren , S. Chen , S. Wilson , K. Nielsch , Q. Y. Zhang , Z. F. Ren , Mater. Today Phys. 2019, 8, 25.

[28] X. Shi , T. Zhao , X. Zhang , C. Sun , Z. Chen , S. Lin , W. Li , H. Gu , Y. Pei , Adv. Mater. 2019, 31, 1903387. 10.1002/adma.201903387 31276253

[29] X. Shi , C. Sun , X. Zhang , Z. Chen , S. Lin , W. Li , Y. Pei , Chem. Mater. 2019, 31, 8987. 10.1002/adma.201903387 31276253

[30] J. Zhang , L. Song , B. B. Iversen , Angew. Chem., Int. Ed. 2020, 59, 4278. 10.1002/anie.201912909 31850591

[31] F. Zhang , C. Chen , S. Li , L. Yin , B. Yu , J. Sui , F. Cao , X. Liu , Z. Ren , Q. Zhang , Adv. Electron. Mater. 2020, 6, 1901391.

[32] Y. Wang , X. Zhang , Y. Liu , Y. Wang , H. Liu , J. Zhang , J. Materiomics 2020, 6, 216.

[33] S. Kim , C. Kim , Y.‐K. Hong , T. Onimaru , K. Suekuni , T. Takabatake , M.‐H. Jung , J. Mater. Chem. A 2014, 2, 12311.

[34] S. Ohno , K. Imasato , S. Anand , H. Tamaki , S. D. Kang , P. Gorai , H. K. Sato , E. S. Toberer , T. Kanno , G. J. Snyder , Joule 2018, 2, 141.

[35] Y. Wang , X. Zhang , Y. Wang , H. Liu , J. Zhang , Phys. Status Solidi A 2019, 216, 1800811.

[36] L. Bjerg , G. K. H. Madsen , B. B. Iversen , Chem. Mater. 2012, 24, 2111.

[37] E. Zintl , E. Husemann , Z. Phys. Chem. 1933, 21B, 138.

[38] L. Song , J. Zhang , B. B. Iversen , J. Mater. Chem. A 2017, 5, 4932.

[39] T. Matsubara , Y. Toyozawa , Prog. Theor. Phys. 1961, 26, 739.

[40] P. E. Blöchl , Phys. Rev. B 1994, 50, 17953. 10.1103/physrevb.50.17953 9976227

[41] G. Kresse , D. Joubert , Phys. Rev. B 1999, 59, 1758.

[42] G. Kresse , J. Furthmüller , Phys. Rev. B 1996, 54, 11169. 10.1103/physrevb.54.11169 9984901

[43] J. Paier , M. Marsman , K. Hummer , G. Kresse , I. C. Gerber , J. G. Angyan , J. Chem. Phys. 2006, 124, 154709. 16674253 10.1063/1.2187006

[44] J. P. Perdew , K. Burke , M. Ernzerhof , Phys. Rev. Lett. 1996, 77, 3865. 10062328 10.1103/PhysRevLett.77.3865

[45] F. Tran , P. Blaha , Phys. Rev. Lett. 2009, 102, 226401. 19658882 10.1103/PhysRevLett.102.226401

[46] S. B. Zhang , J. E. Northrup , Phys. Rev. Lett. 1991, 67, 2339. 10044401 10.1103/PhysRevLett.67.2339

[47] C. G. V. d. Walle , J. Neugebauer , J. Appl. Phys. 2004, 95, 3851.

[48] C. Freysoldt , J. Neugebauer , C. G. Van de Walle , Phys. Rev. Lett. 2009, 102, 016402. 19257218 10.1103/PhysRevLett.102.016402

[49] A. Le Bail , Powder Diffr. 2005, 20, 316.

[50] V. Petricek , M. Dusek , L. Palatinus , Z. Kristallogr. 2014, 229, 345.

[51] L. J. van der Pauw , Philips Res. Rep. 1958, 13, 1.

[52] K. A. Borup , E. S. Toberer , L. D. Zoltan , G. Nakatsukasa , M. Errico , J.‐P. Fleurial , B. B. Iversen , G. J. Snyder , Rev. Sci. Instrum. 2012, 83, 123902. 23278000 10.1063/1.4770124

[53] K. A. Borup , J. de Boor , H. Wang , F. Drymiotis , F. Gascoin , X. Shi , L. Chen , M. I. Fedorov , E. Muller , B. B. Iversen , G. J. Snyder , Energy Environ. Sci. 2015, 8, 423.

[54] S. Iwanaga , E. S. Toberer , A. LaLonde , G. J. Snyder , Rev. Sci. Instrum. 2011, 82, 063905. 21721707 10.1063/1.3601358

[55] M. T. Agne , K. Imasato , S. Anand , K. Lee , S. K. Bux , A. Zevalkink , A. J. E. Rettie , D. Y. Chung , M. G. Kanatzidis , G. J. Snyder , Mater. Today Phys. 2018, 6, 83.

[56] H. Wang , W. D. Porter , H. Böttner , J. König , L. Chen , S. Bai , T. M. Tritt , A. Mayolet , J. Senawiratne , C. Smith , F. Harris , P. Gilbert , J. Sharp , J. Lo , H. Kleinke , L. Kiss , J. Electron. Mater. 2013, 42, 1073.

[57] H. Wang , W. D. Porter , H. Böttner , J. König , L. Chen , S. Bai , T. M. Tritt , A. Mayolet , J. Senawiratne , C. Smith , F. Harris , P. Gilbert , J. W. Sharp , J. Lo , H. Kleinke , L. Kiss , J. Electron. Mater. 2013, 42, 654.

