# Supplementary material for: Probing Efficient N‐Type Lanthanide Dopants for Mg3Sb2 Thermoelectrics
Source: Adv Sci (Weinh). 2020 Nov 13;7(24):2002867. doi: 10.1002/advs.202002867 (PMC7739952; doi:10.1002/advs.202002867)
Supplement: Supplementary file 1 — Supporting Information [file ADVS-7-2002867-s001.pdf]

WILEY-VCH

© 2020 Wiley-VCH GmbH

ADVANCED SCIENCE
Open Access

Supporting Information

for *Adv. Sci.*, DOI: 10.1002/advs.202002867

Probing Efficient n-type Lanthanide Dopants for Mg_3Sb_2 Thermoelectrics

Jiawei Zhang, Lirong Song, and Bo Brummerstedt Iversen**

WILEY-VCH

© 2020 Wiley-VCH GmbH

Supporting Information

Probing Efficient n-type Lanthanide Dopants for Mg_3Sb_2 Thermoelectrics

Jiawei Zhang, Lirong Song, and Bo Brummerstedt Iversen**

Supporting Information

Probing efficient n-type lanthanide dopants for Mg_3Sb_2 thermoelectrics

Jiawei Zhang, Lirong Song, and Bo Brummerstedt Iversen**

Including:

Figures S1-S21

Tables S1-S5

Notes S1-S2

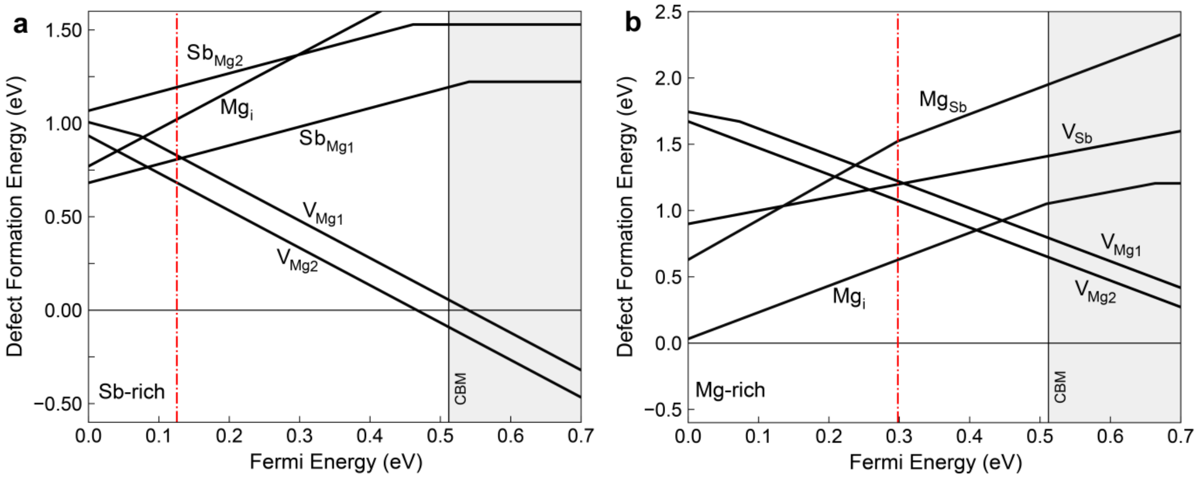

Figure S1. Defect formation energies of native defects in Mg_3Sb_2 under (a) the Sb-rich and (b) Mg-rich conditions. The red dash-dotted line represents the equilibrium Fermi level at the growth temperature of 900 K. The valence band maximum is set to 0 eV. CBM denotes the conduction band minimum.

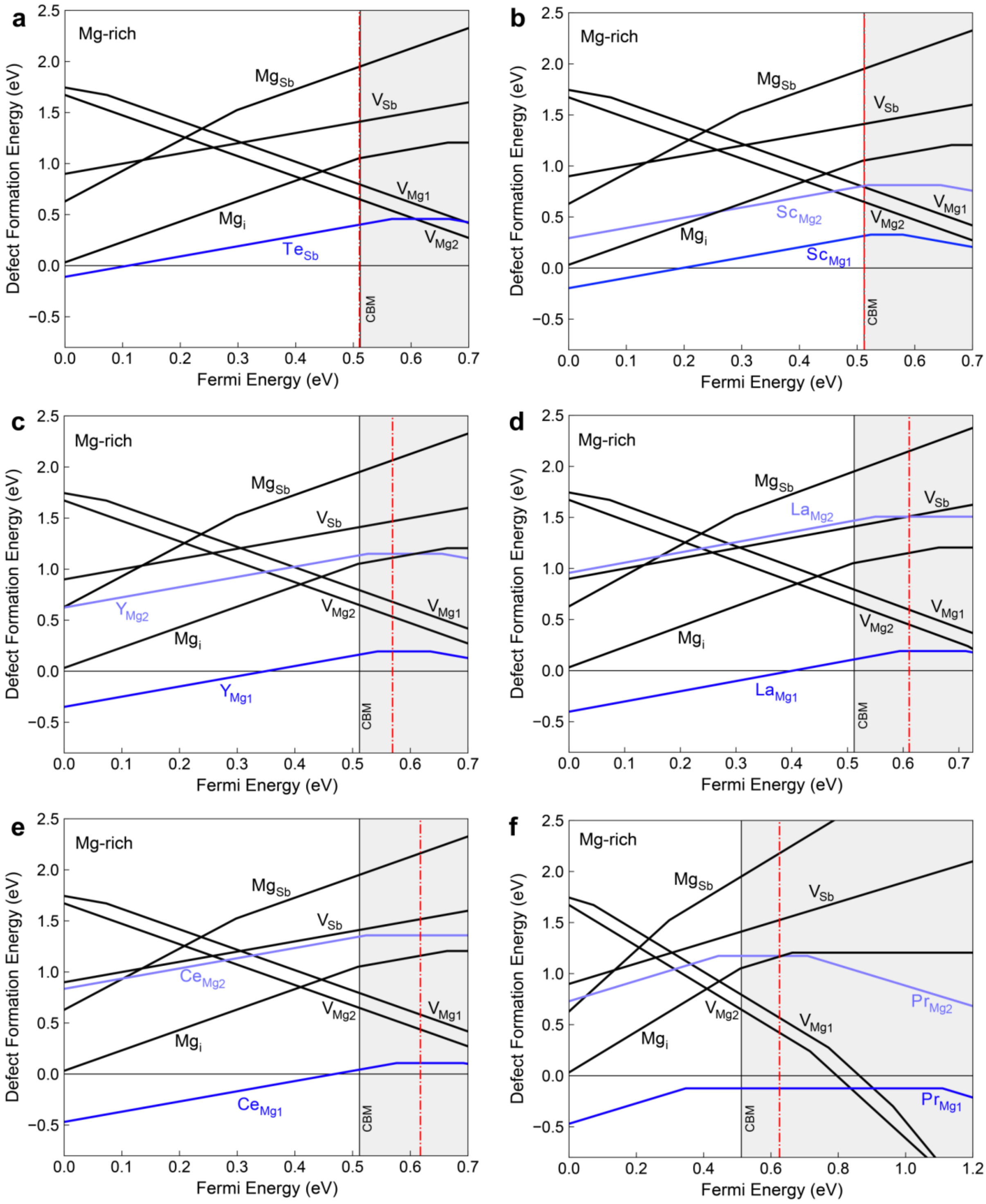

Figure S2. Defect formation energies of (a) the Te doping, (b) Sc doping, (c) Y doping, (d) La doping, (e) Ce doping, and (f) Pr doping in Mg_3Sb_2 under the Mg-rich condition. The red dash-dotted line represents the equilibrium Fermi level at the growth temperature of 900 K. The valence band maximum is set to 0 eV.

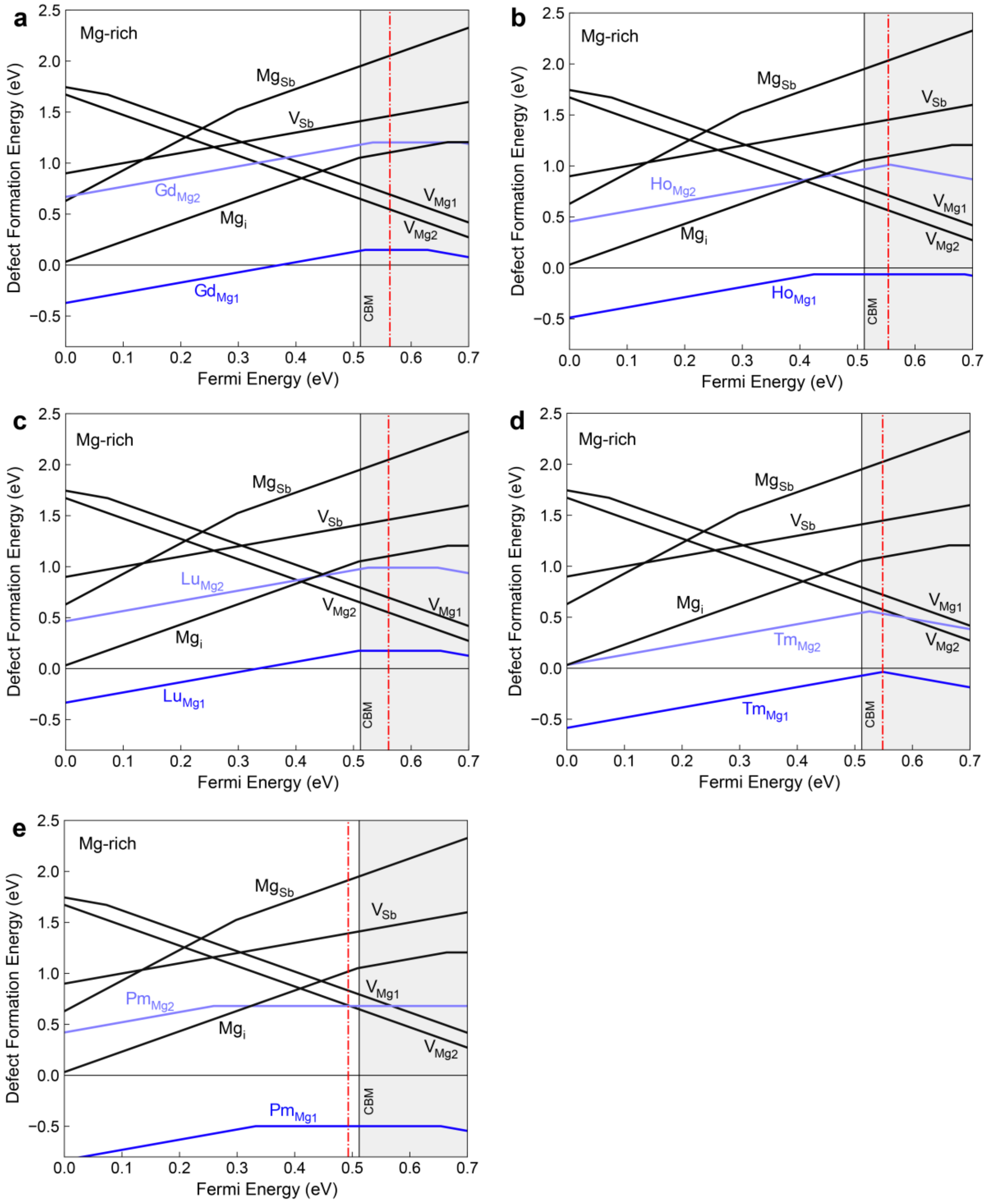

Figure S3. Defect formation energies of (a) the Gd doping, (b) Ho doping, (c) Lu doping, (d) Tm doping, and (e) Pm doping in Mg_3Sb_2 under the Mg-rich condition. The red dash-dotted line represents the equilibrium Fermi level at the growth temperature of 900 K. The valence band maximum is set to 0 eV.

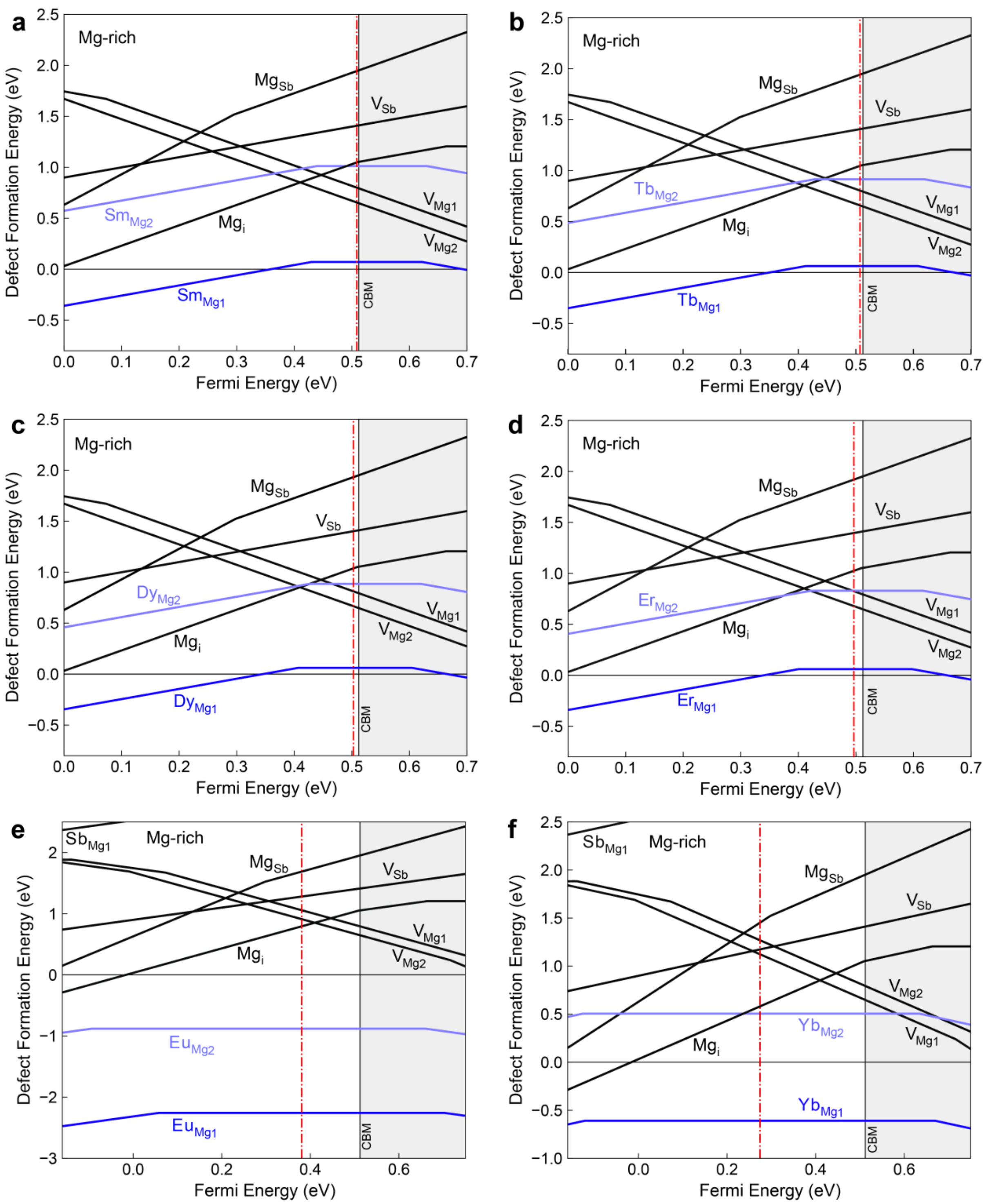

Figure S4. Defect formation energies of (a) the Sm doping, (b) Tb doping, (c) Dy doping, (d) Er doping, (e) Eu doping, and (f) Yb doping in Mg_3Sb_2 under the Mg-rich condition. The red dash-dotted line represents the equilibrium Fermi level at the growth temperature of 900 K. The valence band maximum is set to 0 eV.

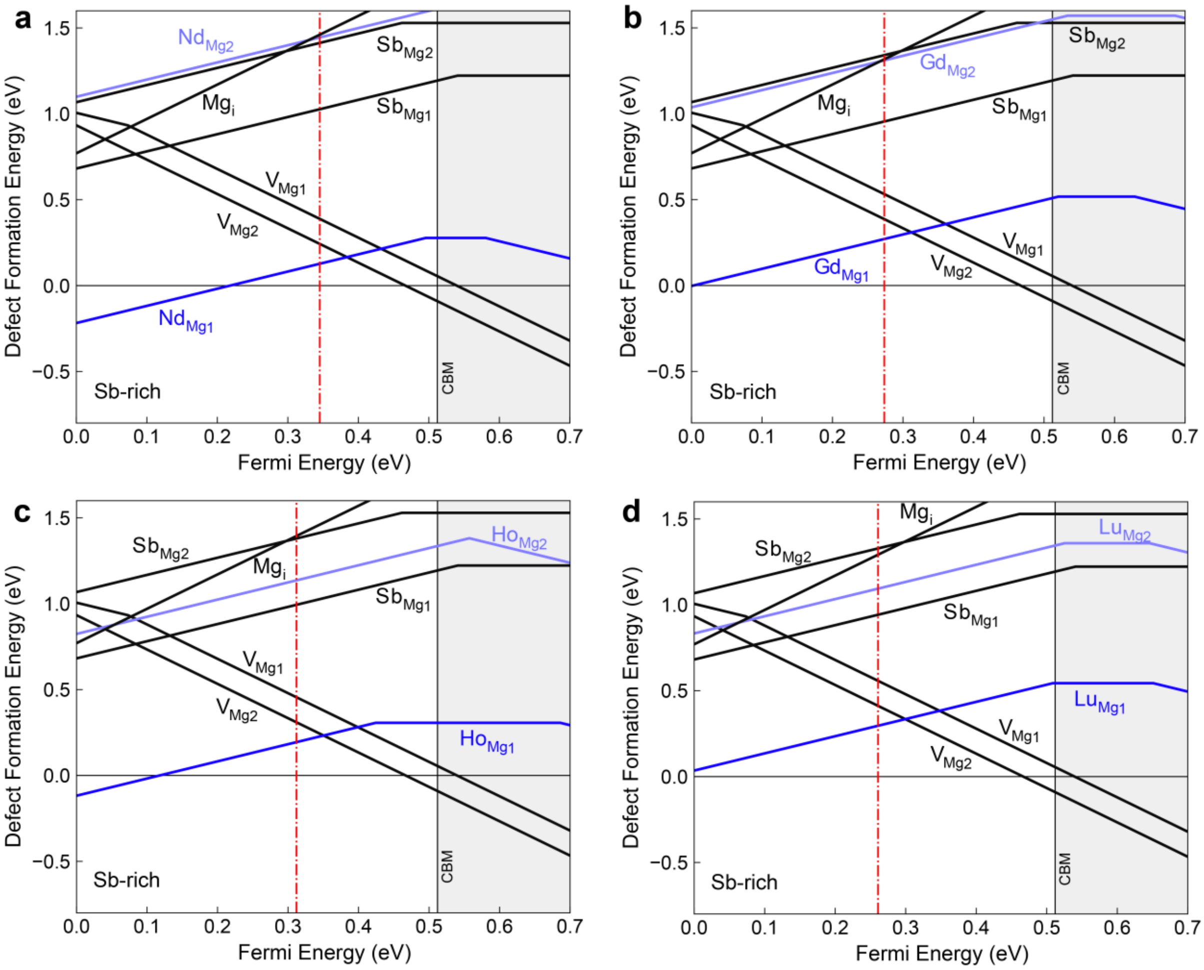

Figure S5. Defect formation energies of (a) the Nd doping, (b) Gd doping, (c) Ho doping, and (d) Lu doping in Mg_3Sb_2 under the Sb-rich condition. The red dash-dotted line represents the equilibrium Fermi level at the growth temperature of 900 K. The valence band maximum is set to 0 eV.

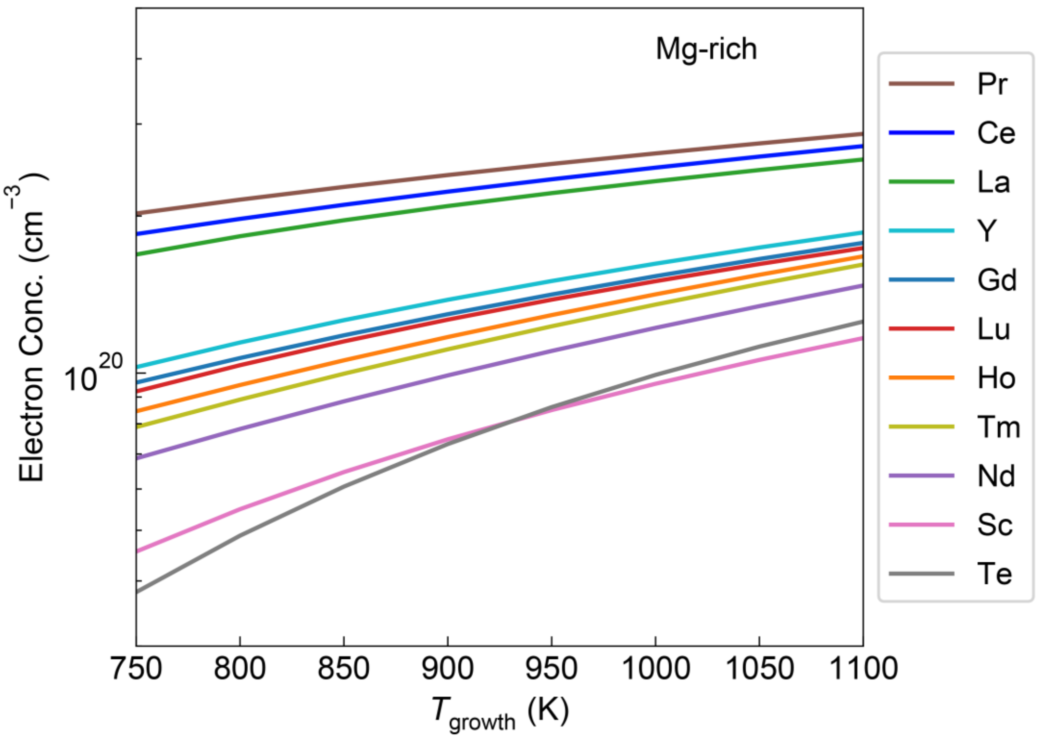

Figure S6. Calculated free electron concentrations *versus* the growth temperature of the efficient n-type doping with lanthanides (i.e., Pr, Ce, La, Gd, Lu, Ho, Tm, Nd) comparing with doping with Te, Sc, and Y in Mg_3Sb_2 under the Mg-rich condition.

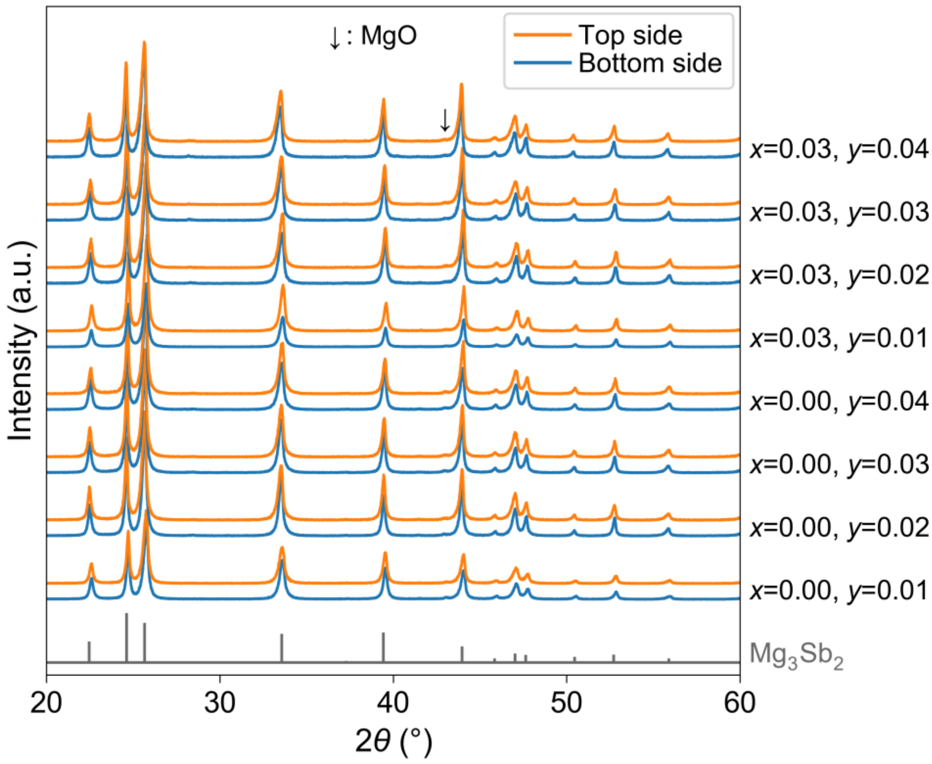

Figure S7. PXRD patterns of the top and bottom sides of the SPS-pressed pellets with the nominal composition $Mg_{3+\delta}Nd_ySb_{2-x}Te_x$ (δ = 0.5, x = 0 and 0.03, y = 0.01-0.04), revealing no clear difference between the top and bottom sides of all samples.

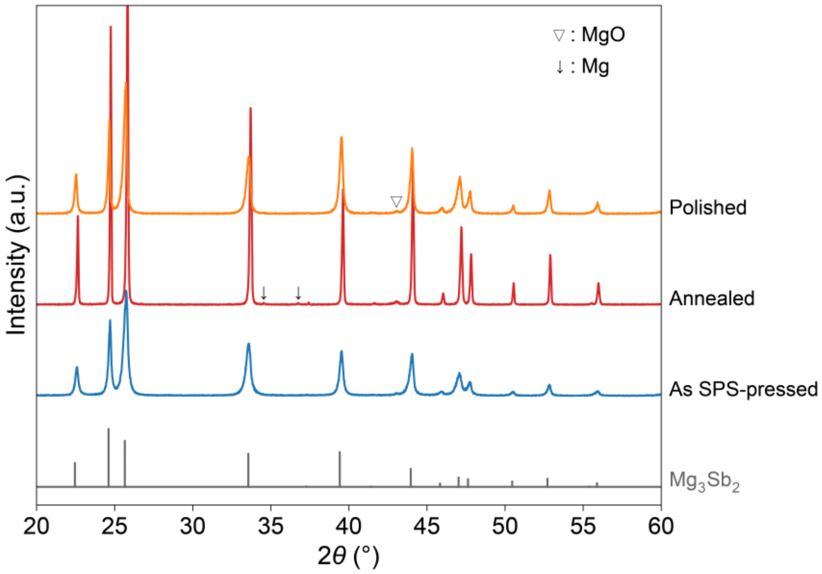

Figure S8. The evolution of the PXRD patterns of the $Mg_{3.5}Nd_{0.01}Sb_2$ pellet showing no clear structural change after annealing in the Mg-rich condition.

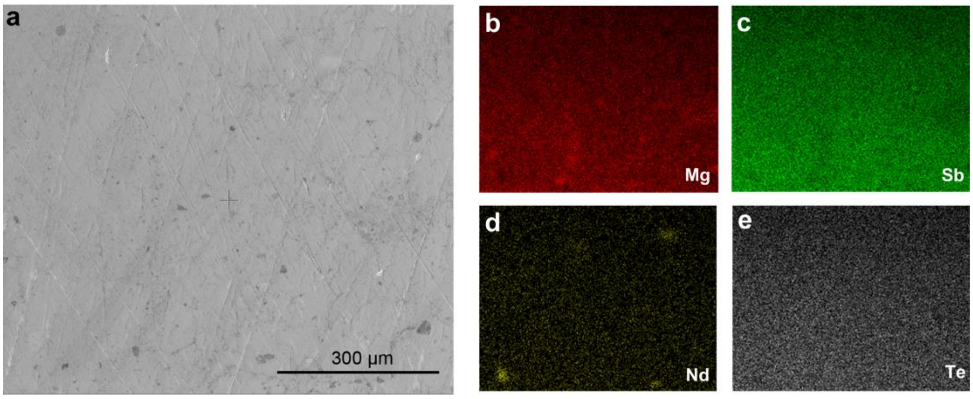

Figure S9. (a) SEM image of the polished surface and (b-e) the corresponding SEM-EDS elemental mapping images of the sample $Mg_{3.5}Nd_{0.03}Sb_{1.97}Te_{0.03}$. The elemental mapping of Nd shows several small Nd-rich regions, which is induced by the presence of a small amount of secondary phase NdSb.

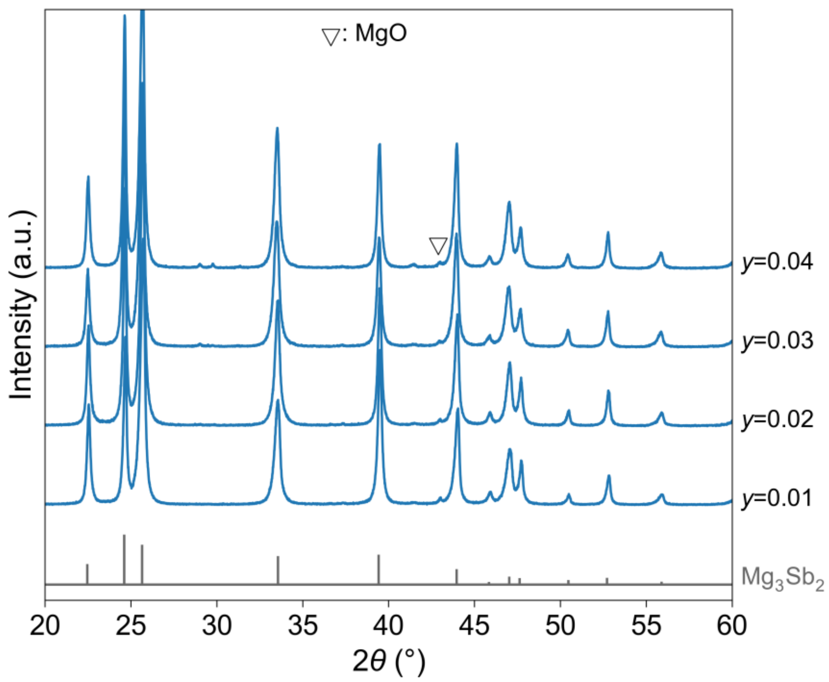

Figure S10. PXRD patterns of n-type $Mg_{3+\delta}Y_ySb_2$ (δ = 0.5, y = 0.01-0.04) samples.

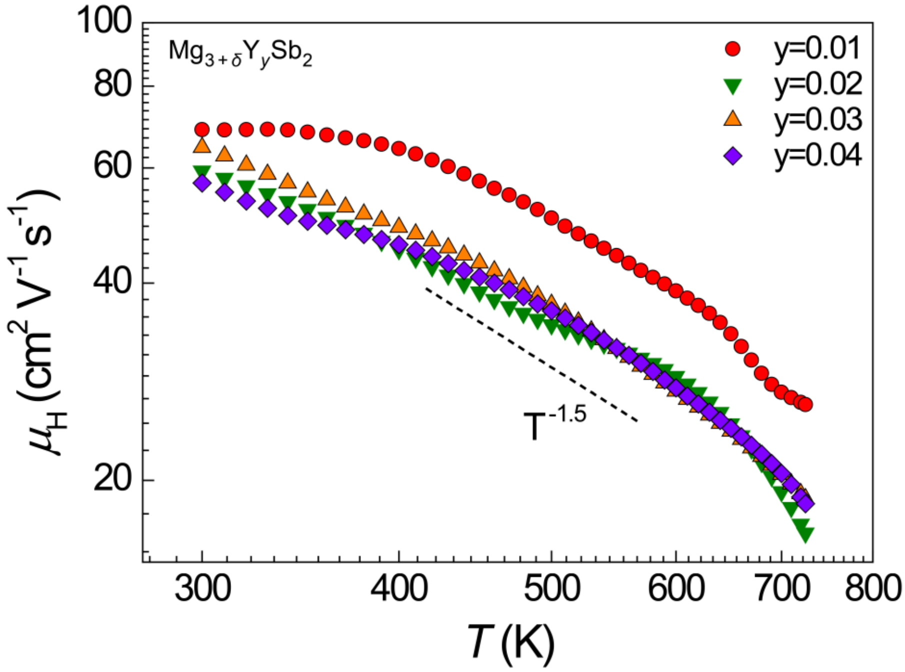

Figure S11. Temperature-dependent electron mobilities of n-type $Mg_{3+\delta}Y_ySb_2$ (δ = 0.5, y = 0.01-0.04) samples.

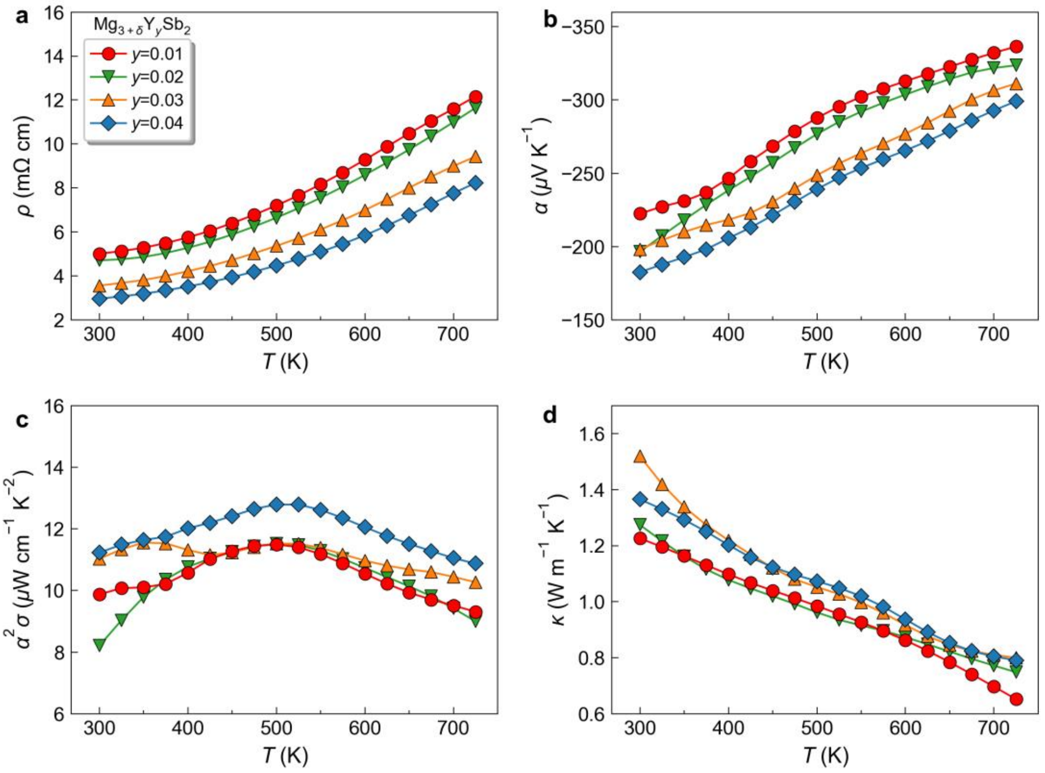

Figure S12. a) Temperature-dependent Seebeck coefficient, b) electrical resistivity, c) power factor, and d) total thermal conductivity of n-type $Mg_{3+\delta}Y_ySb_2$ (δ = 0.5, y = 0.01-0.04).

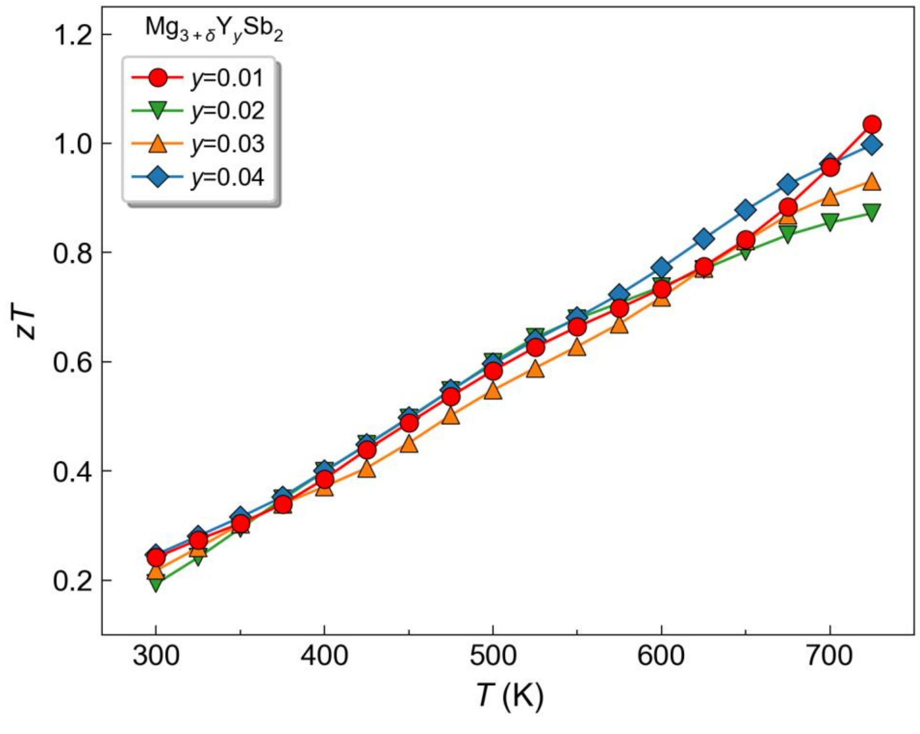

Figure S13. a) Temperature-dependent zT values of n-type $Mg_{3+\delta}Y_ySb_2$ (δ = 0.5, y = 0.01-0.04).

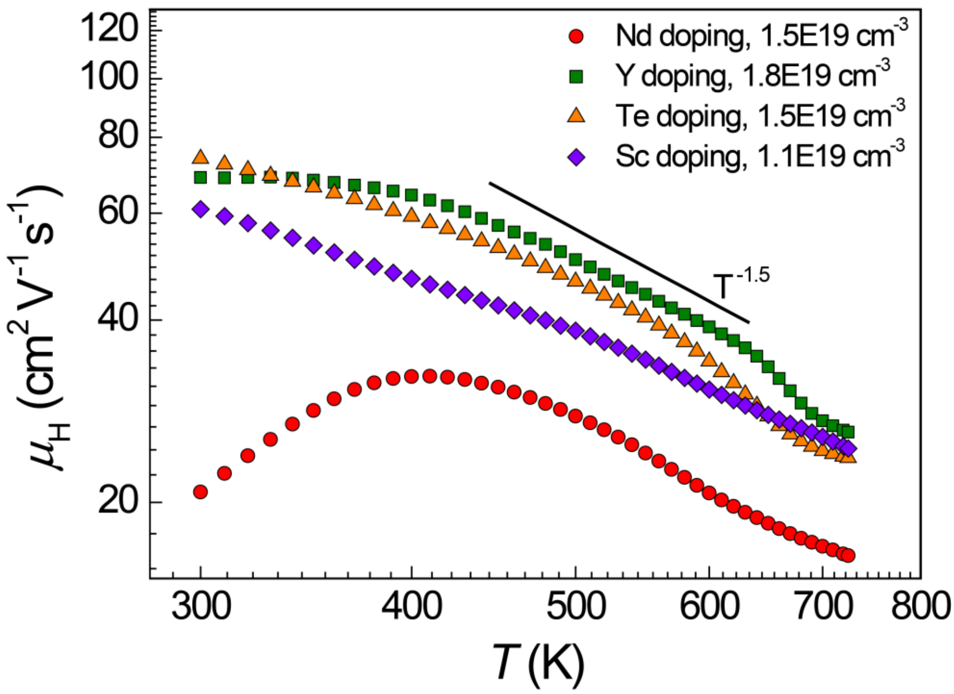

Figure S14. Temperature-dependent electron mobility of n-type $Mg_{3.5}Nd_{0.01}Sb_2$ in comparison with those of n-type $Mg_{3.5}Sb_{1.97}Te_{0.03}$,[1] $Mg_{3.5}Sc_{0.1}Sb_2$,[1] and $Mg_{3.5}Y_{0.01}Sb_2$ with comparable electron concentrations.

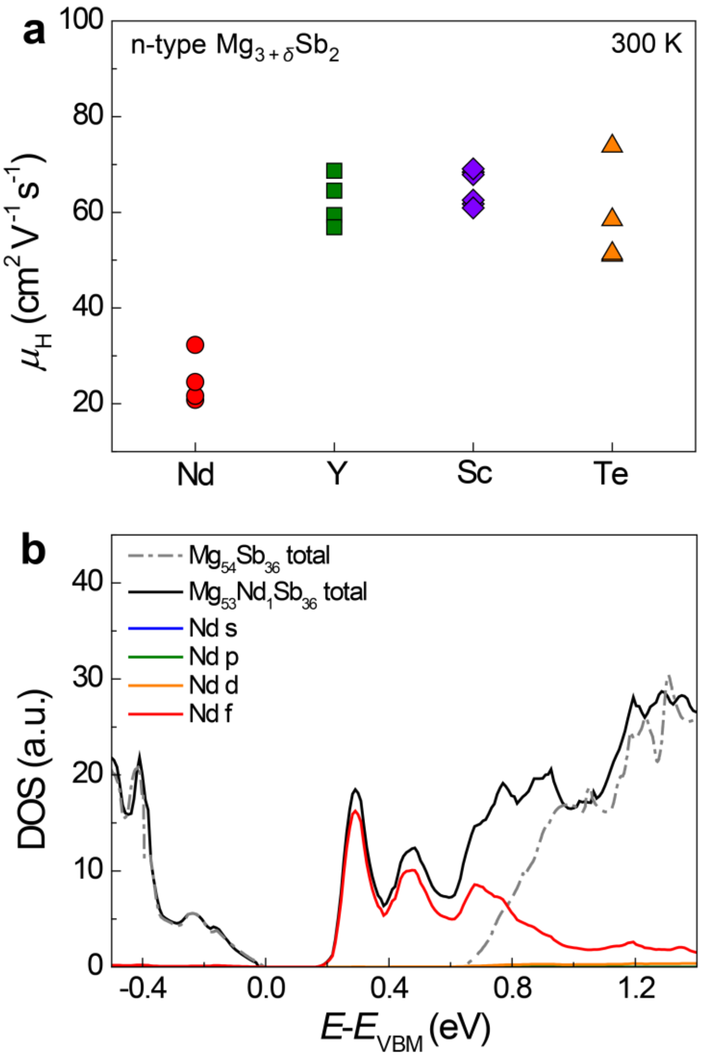

Figure S15. (a) The comparison of room-temperature carrier mobilities of n-type $Mg_{3+\delta}Sb_2$ (δ = 0.5) samples doped with Nd, Y, Sc,[1] and Te.[1] The data of Nd- and Y-doped samples are from this work. (b) The theoretical total density of states (DOS) of $Mg_{53}Nd_1Sb_{36}$ ($Mg_{2.944}Nd_{0.056}Sb_2$) and the partial contributions from the electronic states of Nd. The calculated total DOS of $Mg_{54}Sb_{36}$ (Mg_3Sb_2) is used for comparison.

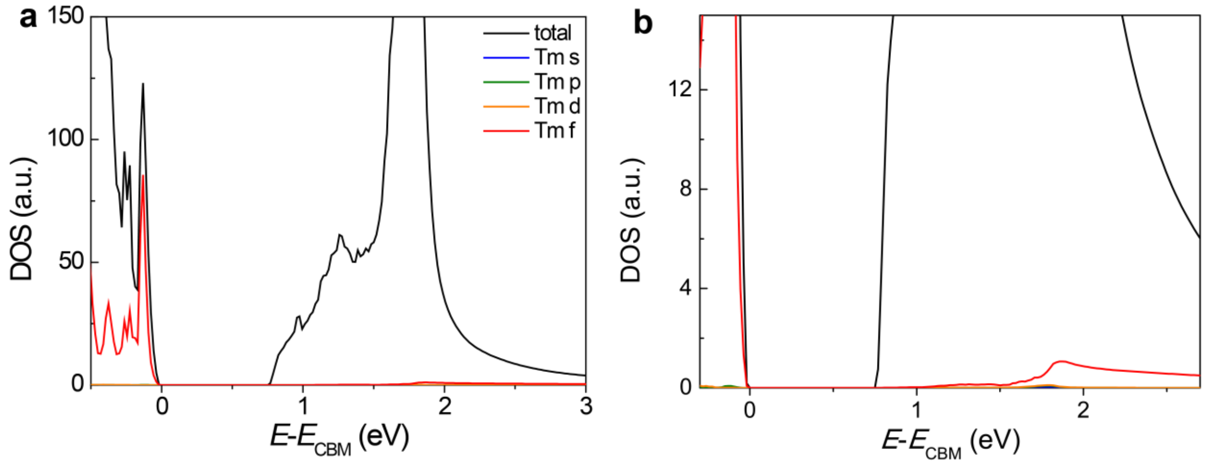

Figure S16. (a) The theoretical total density of states (DOS) and (b) enlarged DOS of $Mg_{53}Tm_1Sb_{36}$ ($Mg_{2.944}Tm_{0.056}Sb_2$) and the partial contributions from the electronic states of Tm.

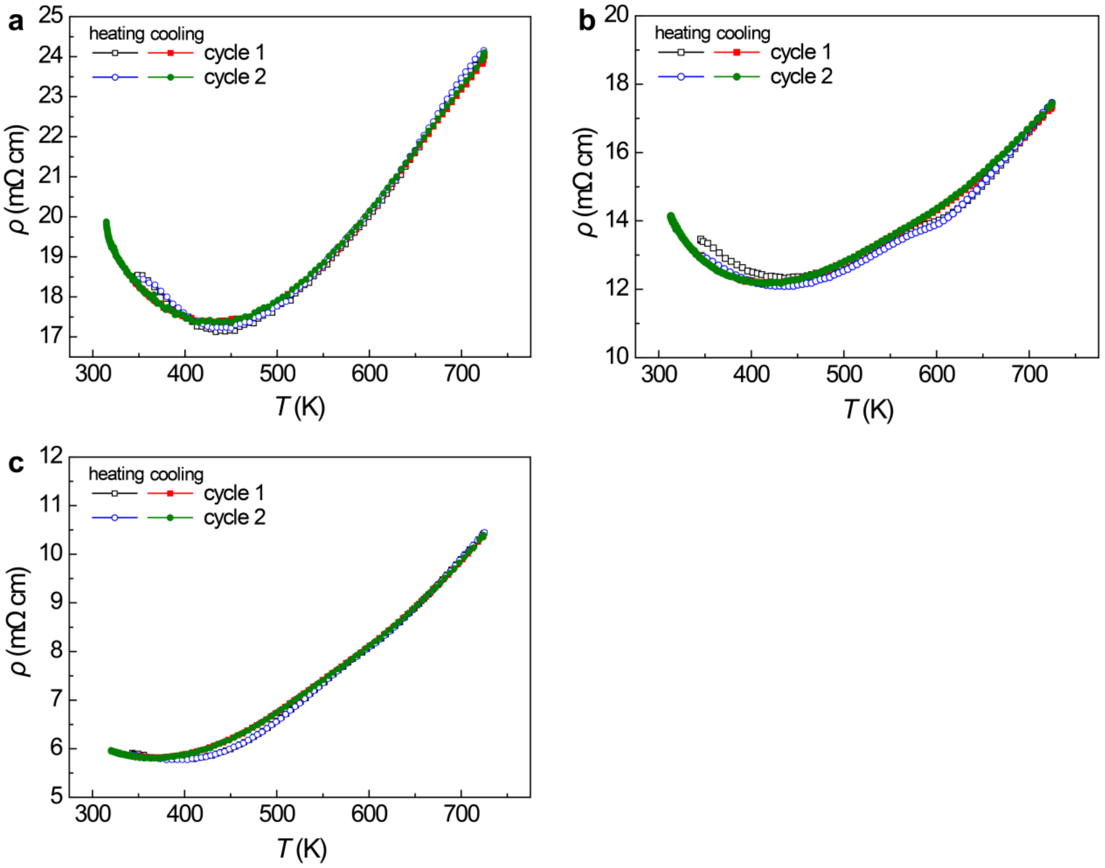

Figure S17. Temperature-dependent electrical resistivity measured upon heating and cooling of the final two stabilized thermal cycles for n-type (a) $Mg_{3.5}Nd_{0.01}Sb_2$, (b) $Mg_{3.5}Nd_{0.02}Sb_2$, and (c) $Mg_{3.5}Nd_{0.03}Sb_2$.

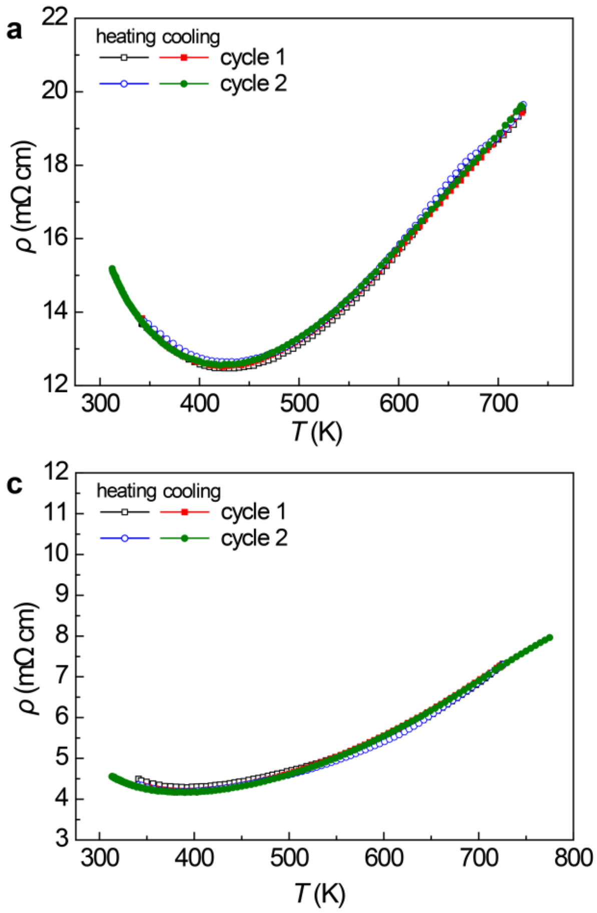

Figure S18. Temperature-dependent electrical resistivity measured upon heating and cooling of the final two stabilized thermal cycles for n-type (a) $Mg_{3.5}Tm_{0.01}Sb_2$, (b) $Mg_{3.5}Tm_{0.02}Sb_2$, (c) $Mg_{3.5}Tm_{0.03}Sb_2$, and (d) $Mg_{3.5}Tm_{0.04}Sb_2$.

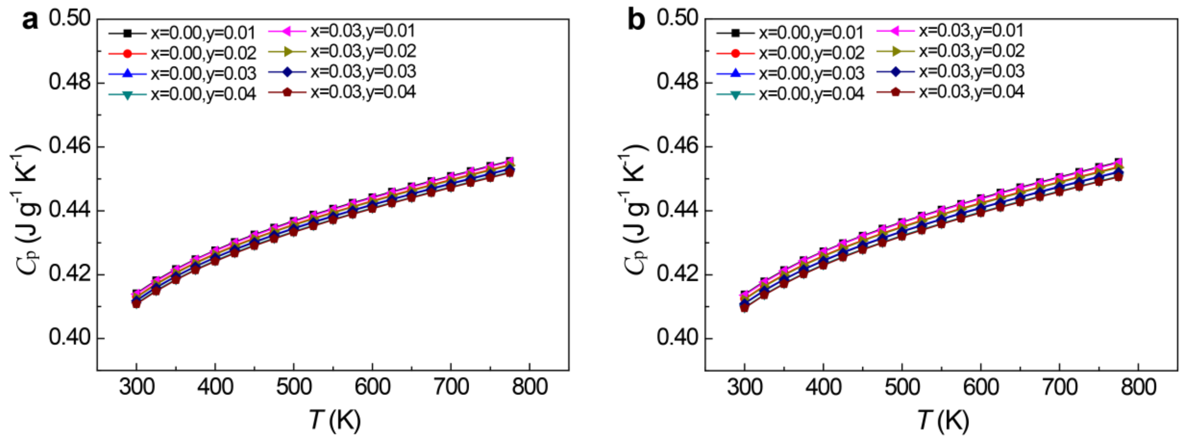

Figure S19. Temperature dependence of heat capacity for n-type (a) $Mg_{3.5}Nd_ySb_{2\text{-}x}Te_x$ and (b) $Mg_{3.5}Tm_ySb_{2\text{-}x}Te_x$ (y = 0.01-0.04, x = 0.00 and 0.03) that were calculated using the polynomial equation proposed by Agne *et al*.[2]

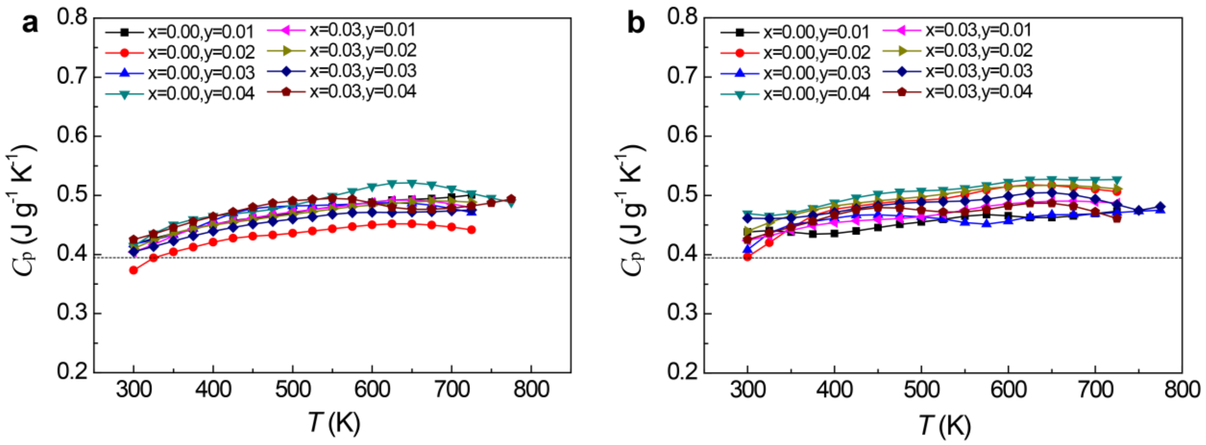

Figure S20. Temperature dependence of heat capacity for n-type (a) $Mg_{3.5}Nd_ySb_{2\text{-}x}Te_x$ and (b) $Mg_{3.5}Tm_ySb_{2\text{-}x}Te_x$ (y = 0.01-0.04, x = 0.00 and 0.03) that were indirectly evaluated by the LFA457 setup. The dashed line represents the Dulong-Petit limit of Mg_3Sb_2.

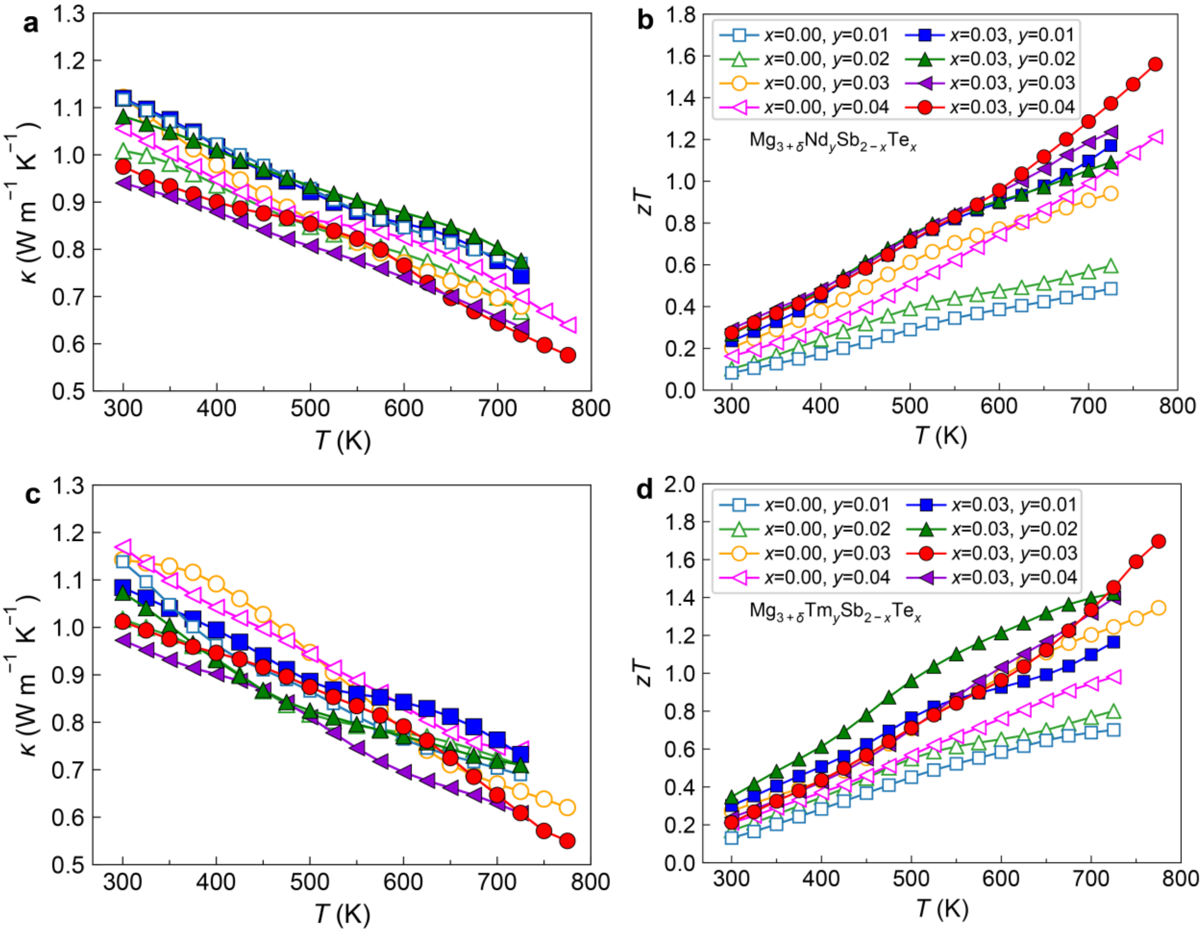

Figure S21. Temperature dependence of total thermal conductivity and *zT* calculated using the heat capacity by the LFA457 setup for n-type (a,b) $Mg_{3.5}Nd_ySb_{2-x}Te_x$ and (c,d) $Mg_{3.5}Tm_ySb_{2-x}Te_x$ (y = 0.01-0.04, x = 0.00 and 0.03).

Table S1. Measured density and relative density of n-type $Mg_{3.5}Nd_{y}Sb_{2-x}Te_{x}$ and $Mg_{3.5}Tm_{y}Sb_{2-x}Te_{x}$ samples.

Samples	Density (g cm^{-3})	Relative density (%)
	$Mg_{3.5}Nd_{y}Sb_{2-x}Te_{x}$	
x=0.00, y=0.01	3.929	97.7
x=0.00, y=0.02	3.953	98.3
x=0.00, y=0.03	3.967	98.7
x=0.00, y=0.04	3.898	97.0
x=0.03, y=0.01	3.887	96.7
x=0.03, y=0.02	3.880	96.5
x=0.03, y=0.03	3.906	97.2
x=0.03, y=0.04	3.876	96.4
	$Mg_{3.5}Tm_{y}Sb_{2-x}Te_{x}$	
x=0.00, y=0.01	3.967	98.7
x=0.00, y=0.02	3.854	95.9
x=0.00, y=0.03	3.932	97.8
x=0.00, y=0.04	3.954	98.4
x=0.03, y=0.01	3.825	95.1
x=0.03, y=0.02	3.935	97.9
x=0.03, y=0.03	3.936	97.9
x=0.03, y=0.04	3.959	98.5

Table S2. Lattice parameters of n-type $Mg_{3.5}Nd_{y}Sb_{2-x}Te_{x}$ and $Mg_{3.5}Tm_{y}Sb_{2-x}Te_{x}$ bulk samples before thermoelectric property measurements.

Samples	a (Å)	c (Å)
	$Mg_{3.5}Nd_{y}Sb_{2-x}Te_{x}$	
x=0.00, y=0.01	4.56461(8)	7.23245(18)
x=0.00, y=0.02	4.56946(10)	7.23858(21)
x=0.00, y=0.03	4.57011(11)	7.23959(22)
x=0.00, y=0.04	4.56699(11)	7.23599(22)
x=0.03, y=0.01	4.56802(11)	7.23924(21)
x=0.03, y=0.02	4.56705(8)	7.23966(17)
x=0.03, y=0.03	4.56814(10)	7.24084(19)
x=0.03, y=0.04	4.56727(9)	7.24088(18)
	$Mg_{3.5}Tm_{y}Sb_{2-x}Te_{x}$	
x=0.00, y=0.01	4.56712(9)	7.23503(18)
x=0.00, y=0.02	4.56909(10)	7.23863(21)
x=0.00, y=0.03	4.56826(10)	7.23786(21)
x=0.00, y=0.04	4.56810(9)	7.23832(18)
x=0.03, y=0.01	4.56689(9)	7.23782(19)
x=0.03, y=0.02	4.56572(8)	7.23731(15)
x=0.03, y=0.03	4.56650(10)	7.23864(18)
x=0.03, y=0.04	4.56701(9)	7.23991(9)

Table S3. The actual composition measured by SEM-EDS for the $Mg_{3.5}Nd_{0.03}Sb_{1.97}Te_{0.03}$ bulk sample before and after annealing in the Mg-rich condition. The result is the average value from the five randomly selected areas of the surface of the pellet.

Samples	Nominal Composition	Composition by SEM-EDS
As SPS-pressed	$Mg_{3.5}Nd_{0.03}Sb_{1.97}Te_{0.03}$	$Mg_{3.05}Nd_{0.03}Sb_{1.95}Te_{0.02}$
After annealing (polished)	$Mg_{3.5}Nd_{0.03}Sb_{1.97}Te_{0.03}$	$Mg_{3.30}Nd_{0.03}Sb_{1.94}Te_{0.02}$

Table S4. The lattice parameters of n-type $Mg_{3.5}Nd_{0.03}Sb_{1.97}Te_{0.03}$ bulk sample before and after annealing in the Mg-rich condition.

Samples	*a* (Å)	*c* (Å)
As SPS-pressed	4.56628(10)	7.23915(19)
After annealing (polished)	4.56814(10)	7.24084(19)

Table S5. Atomic orbital energies of *f* states of lanthanide elements extracted from PAW-PBE pseudopotentials.[3,4] PAW potentials in which the *f* states are treated as valence states were adopted. It should be noted that the atomic orbital energy of Mg 3*s* states is -4.7 eV.

Atoms	Atomic orbital energies of *f* states (eV)
Nd	-6.9
Tm	-9.6
Gd	-8.7
Lu	-9.6
Ho	-9.4
La	-4.4
Ce	-5.4
Pr	-6.2

Note S1. The origin of low electron mobilities in Nd-doped Mg_3Sb_2

At low temperatures, impurity band conduction may happen, where the carrier can propagate within the impurity band without entering the conduction band. If the donors forming the impurity band are partially compensated by acceptors, the band is only partially filled and impurity band conduction can occur within the impurity band.[5] Based on the density of states calculation (see Figure S15), we find that the Nd doping in Mg_3Sb_2 introduces the *f* states of the impurity atom Nd largely below the CBM within the bulk gap, which also show hybridization with the electronic states (Mg 3*s* states) of the near-edge conduction bands resulting in the enhanced DOS of the CBM. This is already obvious with y = 0.056 in $Mg_{3-y}Nd_ySb_2$. These impurity states form impurity band within the bulk gap. From extrinsic defect calculations, we can see that the Nd_{Mg1} defects show both donors (+1) and acceptors (-1) as well as the donor and acceptor transition levels close to the CBM, which is a clear evidence that the Nd_{Mg1} (+1) donor are partially compensated by acceptors within the region near the CBM where the impurity states exist. This indicates that the impurity band conduction may occur in Nd-doped Mg_3Sb_2. As the relatively localized feature of the *f* states, the mobility related to impurity band is generally low due to the heavy dispersion mass, which results in low carrier mobilities at low temperatures.

Note S2. The limitation of the DFT calculations associated with the elements with *f* electrons

It is still a great challenge for presently available density functionals to well handle the systems with *f*-electrons owing to the self-interaction errors.[3] Especially, partially filled *f* states are often incorrectly described, sometimes leading to large uncertainties.[3,4] However, it should be noted that these uncertainties are DFT and not VASP code related. The absolute values of defect formation energies for lanthanide dopants with *f* states by DFT calculations might show some uncertainties and therefore we should focus on the relative values and trends.

References

[1] J. Zhang, L. Song, B. B. Iversen, *Angew. Chem. Int. Ed.* **2020**, *59*, 4278; *Angew. Chem.* **2020**, *132*, 4308.

[2] M. T. Agne, K. Imasato, S. Anand, K. Lee, S. K. Bux, A. Zevalkink, A. J. E. Rettie, D. Y. Chung, M. G. Kanatzidis, G. J. Snyder, *Mater. Today Phys.* **2018**, *6*, 83.

[3] P. E. Blöchl, *Phys. Rev. B* **1994**, *50*, 17953.

[4] G. Kresse, D. Joubert, *Phys. Rev. B* **1999**, *59*, 1758.

[5] T. Matsubara, Y. Toyozawa, *Prog. Theor. Phys.***1961**, *26*, 739.
